# Design and Synthesis of Novel Antileishmanial Compounds

**DOI:** 10.1155/2015/302723

**Published:** 2015-01-21

**Authors:** Melanie Loedige

**Affiliations:** Institute of Organic Chemistry, University of Würzburg, Am Hubland, 97074 Würzburg, Germany

## Abstract

According to the WHO, infectious diseases, and in particular neglected tropical diseases in poor developing countries, still play a significant role in a vast number of deaths reported worldwide. Among them, leishmaniasis occurs as a complex and clinically diverse illness caused by protozoan *Leishmania* species which are transmitted through the bite of sandflies. They develop through a complex life cycle, from promastigotes in sandflies to amastigotes in humans. The severity of disease is determined by the type of infecting *Leishmania* species and also depends strongly on whether the parasite infection leads to a systemic involvement or not. Since the sensitivity towards diverse medicaments highly differs among the *Leishmania* species, it is advantageous to treat leishmaniasis with species-specific drugs. Towards this goal we report a synthetic methodology and characterization of novel small molecular agents active against both forms of *L. major*. This synthetic approach allows for rapid access to new active antileishmanial drug templates and their first derivatives in moderate to very good yields. Although the compounds reported here are bioactive, the detailed biological results are part of a more comprehensive study and will be reported separately by our collaborators.

## 1. Introduction

Infectious diseases are still a major concern worldwide [1, 2]. In spite of the improved living conditions and advances in drug therapy leading to an increased protection from pathogen caused illnesses in industrialized countries, the ongoing research for new drugs is particularly important due to development of resistance to microorganisms [3], creation of new types of pathogenic agents, and the ease of transmission by globalization [2]. In the poor developing nations, which make up almost half of the world population, these kinds of diseases wreak havoc, eventually resulting in death [2]. The available medicaments against the so-called neglected tropical diseases such as leishmaniasis are often either harmful because of their side effects [4], not sufficiently effective, or expensive [5].

**Figure 1:**
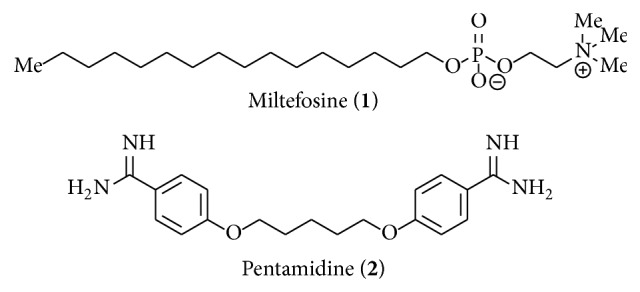
The antileishmanial drugs miltefosine (**1**) and pentamidine (**2**) used as reference drugs in the bioactivity study against* L*.* major*.

Leishmaniasis is caused by about 20 different protozoan* Leishmania* species [6] which are transmitted to humans through the bite of female phlebotomine sandflies [7, 8]. During the dimorphic life cycle, the parasites develop in the guts of the sandfly into promastigotes [9]. Once the parasite is transmitted to humans, they grow up within the parasitophorous vacuole of human macrophages into amastigote forms [10], followed by host cell destruction to release* Leishmania* parasites for repeated infection of macrophages [11]. The severity of disease is in general determined by the grade of systemic involvement and in particular by the type of infecting* Leishmania* species [12, 13]. The clinical picture shows diverse forms of leishmaniasis (visceral leishmaniasis, mucocutaneous leishmaniasis, and various cutaneous forms [6]). With no effective vaccines [14] and no prophylactic treatments currently available [15], with the development of resistance against the established drugs [16], and with irreversible and life-threatening side effects, leishmaniasis certainly paints a grim picture [6]. Most of the drugs such as stibogluconate, amphotericin B, pentamidine, and paromomycine need to be applied parenterally which complicates the administration, and miltefosine is the only orally applicable medicament so far (Figure 1) [17].

Currently affecting about 350 million people with estimated 1.3 million new cases reported annually in 98 countries [18, 19] the search for new orally bioavailable and cost-effective medicaments with good toxicity profiles against leishmanial infections is extremely important [20].

Therefore, we have reported here the synthesis and characterization of novel small molecular compounds such as molecules** 5**,** 11**,** 12**, and** 15** as potential lead structures for the treatment of leishmaniasis (*L*.* major*). The synthetic approach described here has the advantage of being amenable to rapid synthesis of novel antileishmanial drug templates and their derivatives. Although the new molecules reported in this work showed good biological activity against both forms of* L*.* major* in the range of the standard drugs miltefosine and pentamidine or even better, the detailed biological results are part of a more comprehensive study and will be reported separately by our collaborators [21].

## 2. Results and Discussion

### 2.1. Background

**Figure 2:**
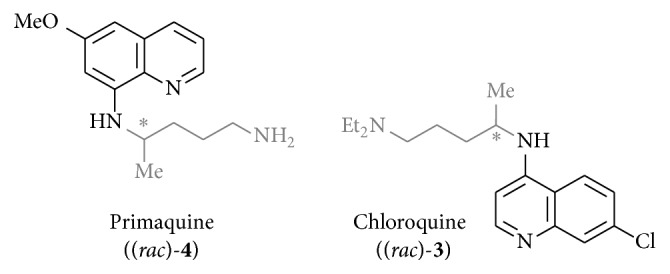
Structures of primaquine ((*rac*)-**4**) and chloroquine ((*rac*)-**3**); in black the corresponding 4-amino-7-chloroquinoline moiety and the 6-methoxy-8-aminoquinoline moiety.

Known heterocyclic structures such as quinolines in general [23, 24], 4-amino-7-chloroquinoline moieties [25] with, for example, chloroquine (**3**) [26], and also 6-methoxy-8-aminoquinolines with primaquine (**4**) [27–31] as well as other heterocyclic substituents have already been described as antileishmanial active (Figure 2).

**Figure 3:**
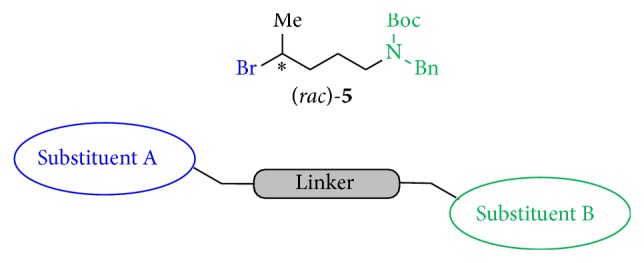
Novel antileishmanial template (*rac*)-**5** and its general scheme.

Thus, our intention towards developing new antileishmanial drugs was to attach variable linkers to these heterocyclic structures which would enable us to link in subsequent synthetic steps other known antileishmanial active heterocyclic moieties. Since the first as linker part synthesized compound (*rac*)-**5** already showed activity in the range of the standard drugs miltefosine (**1**) and pentamidine (**2**), we started to synthesize derivatives of** 5** with varied linker according to chain length (4 and 10 carbon atoms), varied type of linkage (branched and unbranched), different terminal substituents in position A (hydrophobic, hydrophilic, and hydrogen bond forming substituents and aromatic moieties), and also cyclic structures. Eventually, also the structural part which was assumed as pharmacophore (substituent B) of these new templates was analogized (Figure 3).

### 2.2. Chemistry

#### 2.2.1. General Synthetic Procedures

**Scheme 1:**
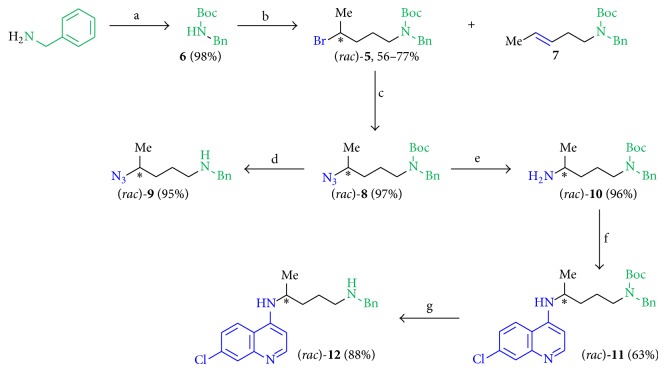
Synthesis of derivatives with a branched alkyl side chain consisting of 5 carbon atoms (**5**,** 7** to** 10**) and the 4-amino-7-chloroquinolinyl substituted derivatives (**11** and** 12**) with antileishmanial activity. Reagents and conditions: (a) Boc_2_O, MeCN (dry), 0–25°C; (b) (*rac*)-1,4-dibromopentane, NaH, DMF (dry), 0–25°C; (c) NaN_3_, DMF (dry), 25°C; (d) TFA, DCM, 25°C; (e) PPh_3_, MeOH (dry), 25°C; (f) 4,7-dichloroquinoline, Pd_2_(dba)_3_, ±-BINAP, KO*t*Bu, 1,4-dioxane (dry), 85°C; (g) TFA, DCM, 25°C.

**Figure 4:**
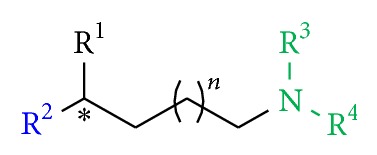
General structure of novel antileishmanial active compounds that were synthesized in this work.

All synthesized novel antileishmanial compounds were based on the plain building block (*rac*)-**5 **(Scheme 1) and their general structure with various R^1^, R^2^, R^3^, and R^4^ substituents which are shown in Figure 4.

All derivatives were synthesized from commercially available precursors purchased from Sigma Aldrich with different chain lengths according to standard chemical procedures, followed by several conversion steps to obtain the derivatives needed for structure activity relationship studies. The general synthetic procedures are as follows.

**Scheme 2:**
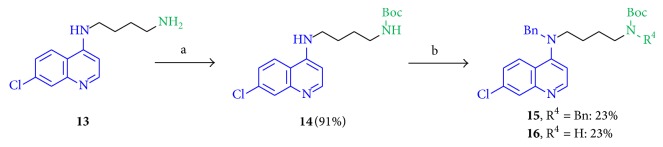
Synthesis of the compounds** 15** and** 16** with additionally benzylated aromatic amine functions. Reagents and conditions: (a) Boc_2_O, MeCN (dry), 0°C; (b) benzyl bromide, NaH, DMF (dry), 0–25°C.

**Scheme 3:**
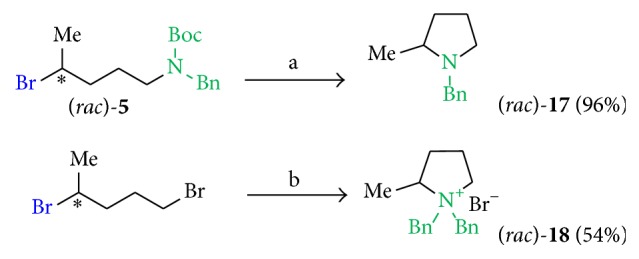
Synthesis of the cyclized products (*rac*)-**17** and (*rac*)-**18** for structure activity relationship studies. Reagents and conditions: (a) TFA, DCM, 25°C; (b) dibenzylamine, acetone, 60°C.

**Scheme 4:**
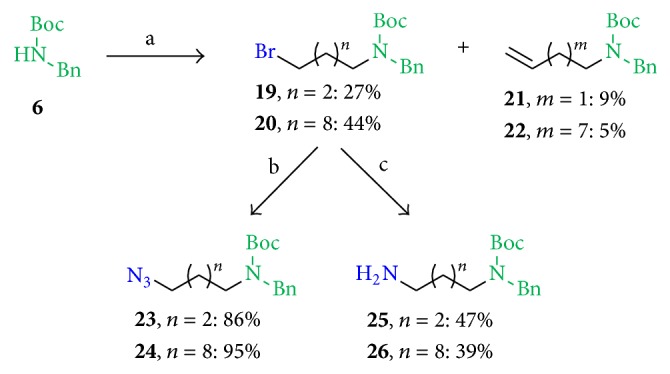
Synthesis of four- and 10-carbon-atom-long unbranched derivatives (**19** to** 26**). Reagents and conditions: (a) 1,4-dibromobutane or 1,10-dibromodecane, NaH, DMF (dry), 0–25°C; (b) NaN_3_, DMF (dry), 25°C; (c) 1: NaN_3_, PPh_3_, 2: KOH, DMF (dry), 25°C.

The primary amine function of benzylamine (Scheme 1) was reacted with Boc_2_O in MeCN (dry) or 3,3-dimethylbutanoyl chloride in DCM (dry) at temperatures from 0°C up to 25°C to give the respective protected starting materials. Deprotonation with NaH in DMF (dry) at 0°C and subsequent reaction with (*rac*)-1,4-dibromopentane (Schemes 1 and 2), 1,4-dibromobutane, and 1,10-dibromodecane (Scheme 3) at 0°C to 25°C gave the bromine derivatives and their elimination products. The terminal bromine atom was used for the introduction of different nitrogen containing substituents such as azide, amine, and heterocycles by nucleophilic substitution reactions (Scheme 1).

In general, the bromine compounds were converted to azides using NaN_3_ in DMF (dry) at room temperature with subsequent reduction to the corresponding amines by Staudinger reaction using a two-step synthetic protocol. The bromine and also the amine function were coupled with different heterocyclic moieties by nucleophilic substitution reactions.

In order to investigate which functional groups of the assumed pharmacophore were required for activity, analogs with diverse terminal functional groups in position B were synthesized. The original* tert*-butyloxycarbonylbenzylamino group was replaced by a* N*-benzyl-3,3-dimethylbutanamide (Scheme 7) functionality as well as by a phthalimide group (Scheme 8).

**Scheme 5:**
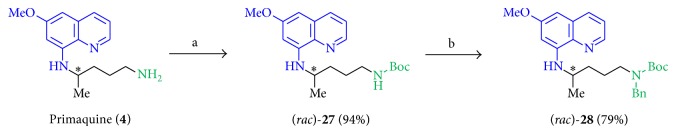
Synthesis of the 6-methoxy-8-aminoquinoline containing derivative (*rac*)-**28**. Reagents and conditions: (a) Boc_2_O, DCM (dry), 0°C; (b) benzyl bromide, NaH, DMF (dry), 0–25°C.

Compounds with a terminal phthalimide functionality in position A were synthesized by the reaction of (*rac*)-1,4-dibromopentane with potassium phthalimide in acetone at 60°C, followed by the introduction of the azide function using NaN_3_ in DMF (dry) at room temperature (Scheme 5).

Cyclized structures were obtained by deprotection of** 5** using TFA in DCM and by reaction of (*rac*)-1,4-dibromopentane with dibenzylamine in acetone at 60°C (Scheme 2).

#### 2.2.2. Results

*(1) Synthesis of the Linker Moiety and the 4-Amino-7-chloroquinoline Substituted Compounds.* For the synthesis of the linker molecule (Figures 3 and 4), we introduced the Boc protecting group to benzylamine according to a literature known protocol [32] using Boc_2_O in MeCN to give compound** 6** in 98% yield, followed by the deprotonation with NaH and by a nucleophilic substitution reaction with 1,4-dibromopentane in DMF (dry) at temperatures from 0°C up to 25°C (Scheme 1, step b). The major and the minor products of the reaction were (*rac*)-**5** and the elimination product** 7**. The yield of the bromine compound (*rac*)-**5** depended on the following factors: (a) the equivalents of NaH, (b) temperature, (c) reaction time, and (d) the total amount of substance in the reaction mixture. Compound (*rac*)-**5** was obtained in varying yields from 56% to 77%, also obtaining elimination product** 7**. Interestingly, since the biological investigation showed that compound (*rac*)-**5** was already antileishmanial active in the concentration range of the reference substances miltefosine (**1**) and pentamidine (**2**), we studied the structure activity relationships of those simple linkage molecules in more detail. The linker molecule was varied by the successful introduction of the azide functional group by using NaN_3_ in DMF (dry) at room temperature to give compound (*rac*)-**8** in 97% yield (Scheme 1, step c). (*rac*)-**8 **was further structurally modified in two ways: (a) by reacting with TFA in DCM at room temperature to remove the Boc protecting group to (*rac*)-**9 (**95%, Scheme 1, step d) and (b) by Staudinger reaction using PPh_3_ in MeOH (dry) at room temperature yielding in amine (*rac*)-**10** (96%, Scheme 1, step e). The free amine function of (*rac*)-**10 **was essential for the coupling to 4,7-dichloroquinoline by a Buchwald-Hartwig amination reaction protocol using Pd_2_(dba)_3_ as catalyst, the ligand ±-BINAP with basic KOtBu in 1,4-dioxane (dry) at 85°C, to give product (*rac*)-**11 **in 63% (Scheme 1, step f), which was Boc deprotected to substance (*rac*)-**12** in 88% yield (Scheme 1, step g).

Derivatives with a tertiary aromatic amine function in position A and with two differently substituted terminal nitrogen atoms in position B were produced (Scheme 2). The Boc group was introduced to obtain** 14** and then reacted with NaH followed by the benzyl bromide addition in DMF (dry) to give compounds** 15** (23%) and** 16** (23%) with the additionally benzylated aromatic amine function.

Since the bromine compound (*rac*)-**5** showed antileishmanial activity, we checked if an* in situ* Boc deprotection and following nucleophilic substitution during the biotests might create (*rac*)-**17** which could have shown activity instead of compound (*rac*)-**5**; thus the synthesis of cyclized derivatives followed (Scheme 3). By using TFA in DCM at room temperature (*rac*)-**5** was converted to an intermediary benzylated amine that instantly cyclized to (*rac*)-**17** in 86% yield. A similar but a quaternary amine compound (*rac*)-**18** (54%) was obtained by using (*rac*)-1,4-dibromopentane and dibenzylamine in acetone at 60°C.

*(2) Variation of the Linker and of Substituents in Position A.* Since we assumed that the Boc group and the benzyl functionality attached to the terminal amine function in position B were the pharmacophore moiety, further derivatives with variable linker were synthesized preserving this moiety in position B. The reaction conditions that were previously successful in the synthesis of (*rac*)-**5** from (*rac*)-1,4-dibromopentane did not work for the reaction of compound** 6** with 1,2-dibromopropane, and hence a two-carbon-atom side chain analog of (*rac*)-**5** could not be synthesized for structure activity relationship studies. The influence of the branched side chain with the additional methyl group and the side chain length was moreover investigated by synthesizing analogs with an unbranched side chain and side chain length of four and 10 carbon atoms. Using the reaction conditions previously successful in the synthesis for (*rac*)-**5**, the unbranched starting materials 1,4-dibromobutane and 1,10-dibromodecane gave in general lower yields (Scheme 4). The bromine compounds** 19** and** 20** each were obtained in 27% and 44% yield, respectively, and the elimination products** 21** and** 22** were obtained in 9% and 5% yield, respectively (Scheme 4, step a). The conversion to the corresponding azides by NaN_3_ in DMF (dry) at room temperature gave** 23** (86%) and** 24** (95%) (reaction monitored by NMR spectroscopy, Scheme 4, step b). Using a one-pot reaction procedure, compounds** 19** and** 20** were stirred separately, first with NaN_3_ and PPh_3_ in DMF at room temperature in an* in situ* Staudinger reaction and a final KOH addition step which hydrolyzes the DMF stable intermediary iminophosphorane compounds resulting in the formation of amines** 25** (47%) and** 26** (39%).

*(3) Synthesis of the 6-Methoxy-8-aminoquinoline Substituted Compounds.* Primaquine (**4**) [27] and its derivatives [28, 29] (Sitamaquine [30] and Tafenoquine [31]) have already been described as antileishmanial active. In order to introduce a 6-methoxy-8-aminoquinoline moiety to the assumed pharmacophore, primaquine (**4**) was protected by a Boc group at the terminal amine functional group using Boc_2_O in DCM (dry) at 0°C in 94% yield ((*rac*)-**27**, Scheme 5, step a), followed by the introduction of the benzyl group to complete the assumed pharmacophore moiety using NaH in DMF (dry) and benzyl bromide to give (*rac*)-**28** in 79% yield (Scheme 5, step b).

**Scheme 6:**
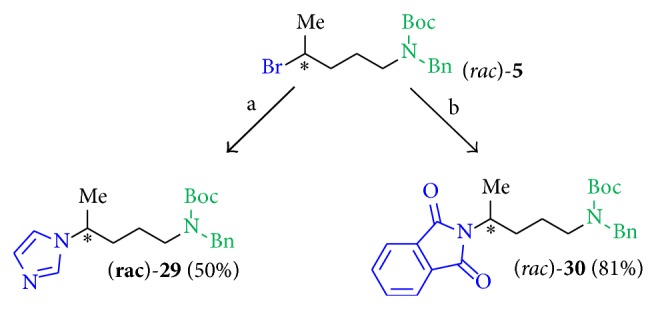
Introduction of additional heterocyclic moieties to the assumed pharmacophore (compounds** 29** and** 30**) for structure activity relationship investigations. Reagents and conditions: (a) imidazole, NaH, DMF, 25°C; (b) potassium phthalimide, DMF (dry), 25°C.

*(4) Linkage to Various Heterocyclic Substituents.* As a monocyclic, aromatic nitrogen containing heterocycle for structure activity relationship studies, imidazole was introduced to the bromine derivative (*rac*)-**5** by using NaH in DMF (dry) to give compound (*rac*)-**29** in 50% yield. Using equal reaction conditions and pyrrole as heterocyclic component, the desired product was not obtained. The introduction of the phthalimide heterocycle using potassium phthalimide in DMF at room temperature gave product (*rac*)-**30** in good yields (81%, Scheme 6).

**Figure 5:**
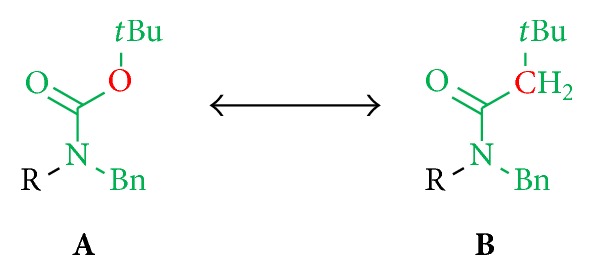
Bioisostere replacement [22] of one oxygen atom (**A**) by a methylene group (**B**).

*(5) Analogs of the Pharmacophore Moiety.* Apart from varying the terminal substituents in position A, we also synthesized structural analogs of the assumed pharmacophore moiety in position B and replaced single atoms in order to investigate the significance of both the Boc and benzyl group in generating antileishmanial activity. Towards this, one oxygen atom of the Boc group was replaced by a carbon atom to examine the effect of the decreased free rotatability of the* tert*-butyl residue and the loss of the hydrogen bond acceptor (Figure 5).

**Scheme 7:**
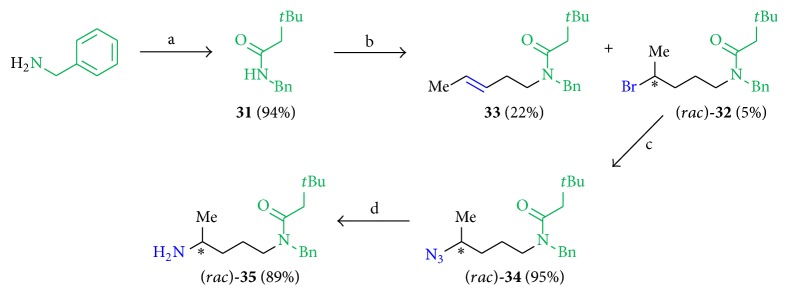
Synthesized analogs (**31** to** 35**) of the assumed pharmacophore moiety. Reagents and conditions: (a) 3,3-dimethylbutanoylchloride, NEt_3_, DCM (dry), 0–25°C; (b) (*rac*)-1,4-dibromopentane, NaH, DMF (dry), 0–25°C; (c) NaN_3_, DMF (dry), 25°C; (d) PPh_3_, MeOH (dry), 25°C.

The key intermediate** 31**, the* N*-benzyl-3,3-dimethylbutanamide, was obtained in 94% yield using benzylamine and 3,3-dimethylbutanoylchloride (Scheme 7) and converted to compound (*rac*)-**32** in very low yields of 5% using NaH, (*rac*)-1,4-dibromopentane in DMF (dry) at temperatures from 0°C up to 25°C where the elimination product** 33** was obtained as the main product (22%). The introduction of the azide functionality using NaN_3_ in DMF (dry) produced compound (*rac*)-**34** (95%), followed by Staudinger reaction to obtain the amine (*rac*)-**35** (89%), both in very good yields. The intended coupling to 4,7-dichloroquinoline under neat conditions at 120°C failed so far, and a Buchwald-Hartwig amination has not been tried yet but it is expected to be successful.

**Figure 6:**
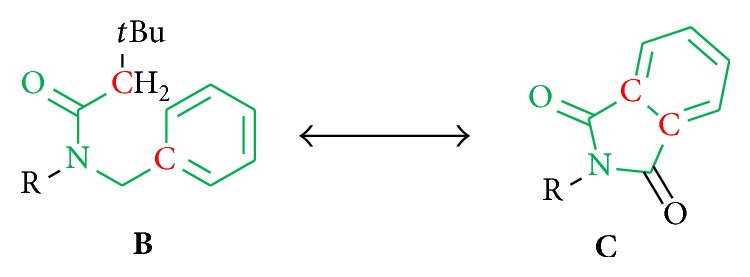
Corresponding components (in green; linkage positions in red) of the motif** B** and the designed compound** C**.

Compounds with a phthalimide group (**36** to** 39**) instead of the assumed pharmacophore moiety in position B were designed as analogs with less steric hindrance and with the benzyl group involved in a fixed ring structure to decrease rotatability (Figure 6).

**Scheme 8:**
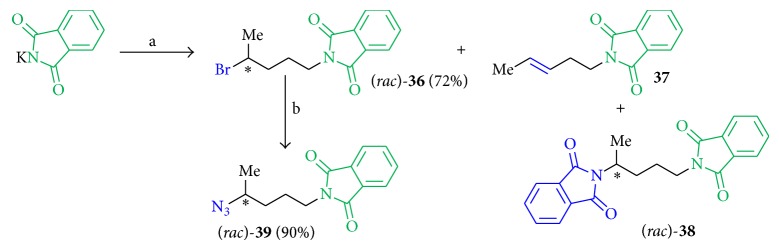
Pharmacophore replacement using a phthalimide motif. Reagents and conditions: (a) (*rac*)-1,4-dibromopentane, acetone (dry), 60°C; (b) NaN_3_, DMF (dry), 25°C.

A reaction between potassium phthalimide and (*rac*)-1,4-dibromopentane [33] in acetone (dry) at 60°C produced not only the bromine derivate** 36** (72%) but also the elimination product** 37 **and the disubstituted compound** 38 **as the minor products. Using NaN_3_ in DMF (dry) gave product** 39** in 90% yield. Because of the lack of biological activity, further derivatization of** 39** was not pursued (Scheme 8).

In summary, the novel pharmacophore type present in the compounds** 6** to** 15 **was investigated for its antileishmanial activity against promastigotes and amastigotes of* Leishmania major*. Since the building block (*rac*)-**5**, previously intended as linkage moiety, already showed activity against* L*.* major* in the concentration range of the reference substances miltefosine (**1**) and pentamidine (**2**), we examined the structure activity relationships of this novel substance class by the synthesis of various analogs.

### 2.3. Discussion

Derivatives of the substance (*rac*)-**5** with variation of both terminal substituents in positions A and B were synthesized to examine the necessary structural elements for antileishmanial bioactivity. The plain starting material compound** 6 **showed no activity at all against promastigotes of* L*.* major* hinting at the requirement of a terminally substituted (position A) alkyl side chain for activity. If the side chain was not substituted as in but-3-enyl-(**21**), dec-3-enyl-(**22**), or pent-3-enyl-residues (**7**), the molecule showed very little to no activity (Table 1). Thus, a nonsubstituted aliphatic side chain does not introduce activity. Antileishmanial activity was observed for a lipophilic bromine substituent (as, e.g., in compounds** 5**,** 19**,** 20**, and** 32**), an azide substituent (as, e.g., in compounds** 8**,** 23**,** 24**, and** 34**), and a terminal amine function (as, e.g., in compounds** 10**,** 25**,** 26**, and** 35**). Compounds (*rac*)-**5**,** 8**,** 20**, and** 26** showed antileishmanial activity values in the same range as the reference substances miltefosine (**1**), the solely orally applicable medicament against leishmaniasis [34], and pentamidine (**2**), with (*rac*)-**5 **showing the highest activity. The cyclization products** 17** and** 18** were inactive (Table 2). If the methyl group in position 4 was lacking, compounds** 19**,** 21**, and** 23** lost activity against promastigotes of* L. major*. The derivatives** 22** and** 24** without a terminal methyl group and with a longer side chain consisting of 10 carbon atoms showed no activity and** 20** and** 26** showed decreased activity (Table 1).

The terminal substituents were varied using a 4-amino-7-chloroquinolinyl (**11** and** 14**), a 6-methoxy-8-aminoquinolinyl (**27 **and** 28)**, a phthalimide (**30**), and an imidazolyl substituent (**29**; Table 2). A larger, lipophilic, nitrogen containing substituent that could build hydrogen bonds and that could cause a trapping of the compound by protonation in acidic compartments of the* Leishmania* organism increased the activity (**11**). For substance** 14** with a higher steric hindrance and higher lipophilic qualities caused by the benzyl group attached to the 4-amino nitrogen atom, the activity compared to compound** 11** decreased. If the same side chain as of** 11** was attached to the quinoline nitrogen atom, compound** 40 **[35] showed also decreased activity. Substituents such as the 6-methoxy-8-aminoquinoline moiety showed no activity at all, and the terminal phthalimide residue** 30** and an imidazolyl substituent as in compound** 29** showed no activity. Similar to** 11**, compound** 29** possessed the imidazolyl moiety which also could be enriched in acidic compartments as described for chloroquine [36] but showed no activity in comparison to the bicyclic derivative** 11**, hinting at the fact that a proton-triggered enrichment in acidic compartments by protonation may not be the main reason for the activity of aminoquinoline substituted compounds. A slightly more spatially demanding substituent was introduced by a nonprotonable phthalimide group, but compound** 30** was not active.

**Figure 7:**
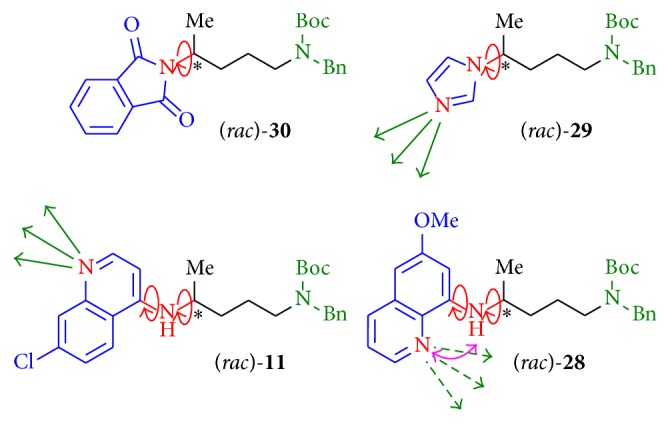
A comparison of rotatability of compounds** 11**,** 28**,** 29**, and** 30**, with diverse terminal substituents.** 29** and** 30** possess only one* C*,*N*-axis (rotatability shown in red curved arrows) in comparison to the two-armed compounds** 11** and** 28**; the quinoline nitrogen atom of compound** 11** could form intermolecular hydrogen bonds (shown as green straight arrows), whereas the rotatability in compound** 28** might be restricted possibly due to the formation of intramolecular hydrogen bond interactions (pink-colored).

The bioactivities vary widely with the diverse terminal substituents and thus it can provide a hint about the nature of very specific interactions with cellular structures of the* Leishmania* species. The ability to build hydrogen bonds seems to be essential for the bioactivity values. The quinoline nitrogen atom of compound** 11** could be accessible to form hydrogen bonds with H-bond donors (Figure 7). In contrast, the basic quinoline nitrogen atom of compound** 28 **has an opposite orientation to that in** 11** and thus could be less capable of building hydrogen bonds. The free rotatability around the* C*,*N*-axes (Figure 7) is probably limited by intramolecular hydrogen bonds which could fix the molecule in a particular conformation.

Due to the free rotatability around two* C*,*N*-axes of compounds** 11** and** 12**, a higher flexibility for the spatial arrangement of the substituents in the target environment might be given compared to compounds** 29** and** 30**. The nitrogen atoms of** 29** and** 30** as well as the quinoline nitrogen atoms of** 11** and** 28** are included as part of the ring structure. The nonincluded NH-function of** 11**, but rather not of** 28**, could contribute as hydrogen bond donor to a possible interaction with the unknown target.

In conclusion, we hypothesize that the interaction of the newly synthesized novel antileishmanial compounds with the unknown target on the protozoan might be influenced by the lipophilicity and size as well as the distance between the substituents in positions A and B, their flexible spatial orientation, and their ability to interact* via* hydrogen bonds.

**Figure 8:**
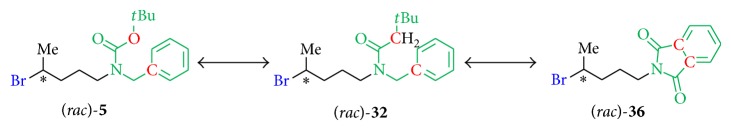
Compound** 5** and its pharmacophore analogs** 32** and** 36**.

To clarify the importance of the original* tert*-butyloxycarbonylbenzylamino substituent for the bioactivity, the four terminal functional groups which were used initially in position A (bromine, azide, amine substituent, and the terminal double bond) were retained, and the assumed pharmacophore moiety in position B was varied (Figure 8).

**Figure 9:**
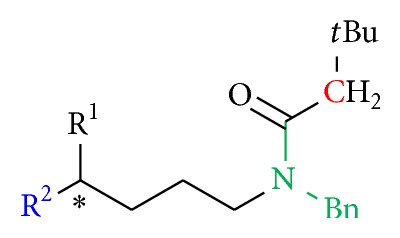
General structure of analogs of the assumed pharmacophore moiety having a* N*-benzyl-3,3-dimethylbutanamide functionality.

**Figure 10:**
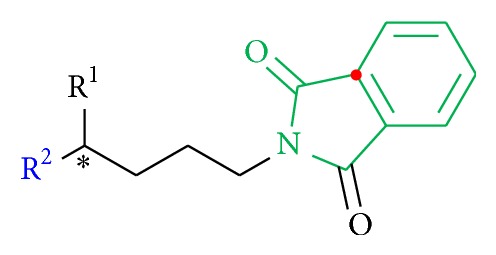
General structure of analogs of the assumed pharmacophore moiety with a phthalimide functionality.

The Boc- and benzyl-substituted amine function was replaced by a* N*-benzyl-3,3-dimethylbutanamide residue (**31**) which resulted in decrease in bioactivity for compound** 32** relative to** 5** and for** 34** in comparison to** 8** (Figures 8 and 9, Table 3). Compounds** 31** and** 6** showed no activity. These observations emphasize the need for hydrogen bond formation and of more freely rotatable bonds around the* C*,*N*-axis. The oxygen atom of the Boc group seems to be essential for the interaction (Figure 8). The introduction of a phthalimide function leads to a drastic decrease in activity for all compounds (**36**,** 37**,** 38**, and** 39**) (Figure 10, Table 4).

We also investigated the activity of both the Boc and benzyl group as in the compounds** 8**/**9**,** 11**/**12**,** 15**/**16**,** 27/28**, and** 40**/**41**. The removal of the Boc group caused a significant loss in activity. Compound** 8** lost all activity by removal of the Boc group (**9**) and substance** 12** with a 4-amino-7-chloroquinolinyl substituent showed a significant decrease in comparison to molecule** 11**. The 6-methoxy-8-aminoquinoline derivatives** 27 **and** 28** showed no activity against promastigotes of* L*.* major*. Similar to the initial observations, the removal of the Boc group from the 4-amino-7-chloroquinolinium salt** 40** leads to a decrease of activity** 41** [35], and loss of the benzyl group leads to an activity loss of** 15** to** 16**. This shows that it is extremely essential for the Boc and benzyl group to be attached to the amine function in order for these novel molecules to show antileishmanial activity against* L*.* major*.

The four most active compounds against promastigotes of* L*.* major* were also tested against amastigotes and showed even higher activity values. The bromine derivative** 5** as a simple representative of the new substance class was active in the same concentration range as the reference substances miltefosine (**1**) and pentamidine (**2**), whereas azide** 8** already showed a slightly higher activity against amastigotes. The most active representatives against promastigotes with a 4-amino-7-chloroquinoline substituent** 11** and** 12** showed higher activity against amastigotes than promastigotes with** 11** showing slightly higher activity while** 12** was significantly more active. The activity values of those compounds against promastigotes may be probably correlated with activities against amastigotes; amastigotes seem to react stronger in the case of the compounds** 5**,** 8**,** 11**, and** 12**. Investigation of bioactivity against* L*.* donovani* in the presence of these novel compounds showed no significant activities for all synthesized compounds, thus showing that these newly synthesized compounds might be highly selectively active against* L*.* major*.

### 2.4. Conclusion

We have synthesized and characterized a novel class of compounds with highly selective activity against* L*.* major*. We also investigated the structure activity relationship against promastigotes of* L. major* and compared the activity values of promastigotes to the obtained activities of amastigotes. Terminal substituents in position A and the assumed pharmacophore moiety in position B were varied.

**Scheme 9:**
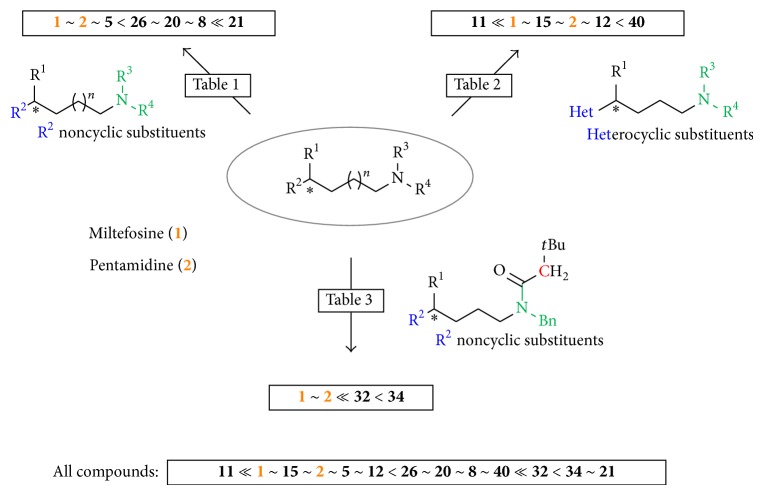
All active molecules of the study and reference drugs miltefosine (**1**) and pentamidine (**2**) in order from highest (**11**; 2.9 times more active than** 1**) to lowest (**21**; 2.8 times less active than** 1**) activity against promastigotes of* L*.* major* (against amastigotes of* L*.* major*:** 12** <** 11** <** 8** <** 5**).

The most active substances were the 4-amino-7-chloroquinolinyl substituted compound** 11**, followed by the* N*-benzylated derivative** 15**, prototype compound** 5,** and the Boc deprotected compound** 12 **(Scheme 9). The bromine substituted prototype substance** 5** showed already good activity against promastigotes and amastigotes of* L*.* major* in the same concentration range as the reference substances miltefosine (**1**) and pentamidine (**2**).

The observed bioactivities and the short synthesis routes provide an excellent method for screening new drug targets for activity against leishmaniasis in general and* L*.* major* in particular.

### 2.5. Experimental Section

#### 2.5.1. General Information

All used solvents were distilled before use. Commercially available material was purchased from Sigma Aldrich and used without further purification. Thin-layer chromatography was carried out using silica gel 60 F_254_ or alumina with fluorescent indicator. Detection of the compounds was achieved by fluorescence quenching at 254 nm, fluorescence at 356 nm, or staining with iodine or ninhydrin. Flash chromatography was performed using silica gel (20–63 mesh), deactivated silica gel (20–63 mesh; 7.5% ammonia), or ICN neutral or basic alumina, deactivated with 15% H_2_O. NMR spectra were obtained on a Bruker DMX 600 apparatus and are reported in ppm relative to internal solvent signal with coupling constants (*J*) in Hertz (Hz). Spectra were usually obtained at 25°C, and compounds** 19–25** and** 26** were measured at (calibrated) 2°C or 3°C, respectively. EI mass spectrometry was carried out on a Finnigan MAT 8200; ESI-HRMS was measured on a Bruker Daltonik micrOTOF-focus.

#### 2.5.2. Synthesis and Characterization of Antileishmanial Compounds

*tert-Butyl Benzylcarbamate (* ***6*** *) [32].* Boc_2_O (43.890 g, 0.201 mol) was added to a solution of benzylamine (19.620 g, 0.183 mol, and 20.0 mL) in MeCN (dry, 100 mL) at 0°C and the reaction mixture was stirred for 3 hours at 25°C. The mixture was concentrated, and the residue was suspended in aqueous NaOH solution and exhaustively extracted using DCM. The combined organic extracts were dried (MgSO_4_), filtered, and concentrated. Compound** 6** (37.022 g, 98%) was obtained as colorless crystals. Mp 56°C (DCM)—IR (ATR-FTIR): $\tilde{\nu }$ = 3338 (w), 3304 (w), 3063 (w), 3035 (w), 2979 (w), 2930 (w), 2361 (w), 2341 (w), 1737 (w), 1701 (w), 1674 (s), 1607 (w), 1587 (w), 1539 (m), 1495 (w), 1452 (m), 1388 (w), 1364 (m), 1328 (w), 1310 (w), 1268 (m), 1252 (m), 1206 (w), 1163 (m), 1136 (m), 1081 (w), 1051 (m), 1027 (m), 1018 (w), 947 (w), 929 (w), 915 (w), 863 (m), 817 (w), 765 (w), 746 (m), 723 (m), 695 (s), 656 (m), 630 (w), 616 (w) cm^−1^—^1^H-NMR (400 MHz, CDCl_3_): *δ* = 1.57 (s, 9 H,* t*Bu-Me), 4.29 (s, 2 H, CH_2_Ph), 4.79 (s, broad, NH), 7.21–7.32 (m, 5 H, Ph-H) ppm—MS (EI, 70 eV): *m*/*z* (%) = 151.1/150.1 [M-C_4_H_9_]^+^ (91/100), 107.1/106.1 [M-C_5_H_9_O_2_]^+^ (5/38), 92.1/91.1 [C_7_H_7_]^+^ (5/63), 58.1/57.1 [C_4_H_9_]^+^ (4/90)—HRMS (ESI) calcd. [M+Na]^+^ 230.11515; found 230.11507.

*tert-Butyl Benzyl(4-bromopentyl)carbamate (* ***5*** *).* NaH (955.2 mg of a 55% oily dispersion, 21.89 mmol) was added in portions to a solution of compound** 6** (1.479 g, 7.14 mmol) in DMF (dry, 30 mL) at 0°C under nitrogen atmosphere and the reaction mixture was stirred for 30 min at 0°C. (*rac*)-1,4-Dibromopentane (3.610 g, 15.70 mmol, and 2140 *µ*L) was added and stirred for 90 min at 0°C and the mixture was allowed to warm up to 25°C. After 5 hours of stirring, the excess of NaH was carefully hydrolysed using water and the mixture was exhaustively extracted with dichloromethane. The combined organic extracts were dried (MgSO_4_), filtered, and concentrated. The solvent residue was removed azeotropically as a mixture with toluene. Purification with flash column chromatography on silica gel (petroleum ether/EtOAc 20 : 1) gave** 5** (1.965 g, 77%) as colorless oil. IR (ATR-FTIR): $\tilde{\nu }$ = 2972 (w, br), 2926 (w, br), 2358 (w, br), 2341 (w, br), 1687 (s), 1495 (w), 1453 (m), 1413 (m), 1391 (w), 1378 (w), 1364 (m), 1282 (w), 1242 (m), 1159 (s), 1134 (s), 1090 (w), 1074 (w), 1049 (w), 1028 (w), 964 (w), 898 (w), 872 (m), 818 (w), 768 (w), 733 (m), 698 (m), 670 (w), 632 (w), 615 (w) cm^−1^—^1^H-NMR (600 MHz, CDCl_3_): *δ* = 1.45 (d, 9 H,* t*Bu-Me), 1.61 (m, 1 H, 2-H), 1.65 (d, ^3^ *J*_H–H_ = 6.66 Hz, 3 H, 5-Me), 1.70–1.72 (m, 3 H, 2-H, 3-CH_2_), 3.12–3.24 (m, 2 H, 1-H), 4.06–4.10 (m, 1 H, 4-H), 4.36–4.42 (m, 2 H, CH_2_Ph), 7.21–7.25 (m, 3 H, Ph-H), 7.29–7.31 (m, 2 H, Ph-H) ppm—^13^C-NMR (150 MHz, CDCl_3_): *δ* = 26.25 (C-2), 26.55 (C-2), 26.68 (Me), 28.64 (*t*Bu-Me), 38.34 (C-3), 45.73 (C-1), 49.98 (NCH_2_Ph), 50.46 (NCH_2_Ph), 51.26 (C-4), 51.62 (C-4), 79.98 (*t*Bu-C), 127.38 (*o*-Ph-CH), 127.95 (*p*-Ph-CH), 128.69 (*m*-Ph-CH), 138.52 (Ph-C), 138.68 (Ph-C), 155.88 (Boc CO), 156.21 (Boc CO) ppm—MS (EI, 70 eV): *m*/*z* (%) = 301.2/300.2/299.2 [M-C_4_H_8_]^+***·***^ (28/7/28), 220 [M-C_4_H_8_-Br]^+^ (15)—HRMS (ESI) calcd. [M+Na]^+^ 378.10391; found 378.10391.

The elimination product* (E)-tert-butyl benzyl(pent-3-enyl)carbamate* (**7**, 375.9 mg, 19%) was obtained as colorless oil. IR (ATR-FTIR): $\tilde{\nu }$ = 2974 (w), 2930 (w), 2360 (w), 2342 (w), 1691 (s), 1605 (w), 1495 (w), 1454 (m), 1411 (m), 1364 (m), 1290 (w), 1242 (m), 1164 (s), 1134 (m), 1074 (w), 1028 (w), 1000 (w), 965 (m), 876 (m), 771 (w), 731 (m), 698 (m), 668 (w), 639 (w), 629 (w), 620 (w), 608 (w) cm^−1^—^1^H-NMR (600 MHz, CDCl_3_): *δ* = 1.41–1.48 (d, 9 H,* t*Bu-Me), 1.61 (d, 3 H, ^3^ *J*_H–H_ = 6.00 Hz, Me), 2.12–2.17 (m, 2 H, 2-H), 3.11–3.21 (m, 2 H, 1-H), 4.38–4.43 (m, 2 H, CH_2_Ph), 5.31–5.41 (m, 2 H, 3-H, 4-H), 7.20–7.23 (m, 3 H,* o*-Ph-H,* p*-Ph-H), 7.28–7.31 (m, 2 H,* m*-Ph-H) ppm—^13^C-NMR (150 MHz, CDCl_3_): *δ* = 18.21 (Me), 28.65 (*t*Bu-Me), 31.43 (C-2), 31.82 (C-2), 46.71 (C-1), 46.83 (C-1), 50.12 (CH_2_Ph), 50.82 (CH_2_Ph), 79.65 (*t*Bu-C), 79.78 (*t*Bu-C), 127.21 (C-4), 127.27 (*o*-Ph-CH), 127.92 (*p*-Ph-CH), 128.11 (C-3), 128.62 (*m*-Ph-CH), 138.67 (Ph-C), 138.94 (Ph-C), 155.75 (Boc CO), 156.26 (Boc CO) ppm—MS (EI, 70 eV): *m*/*z* (%) = 221.2/220.2 [M-C_4_H_7_]^+^ (4/29), 165.1/164.1 [M-C_8_H_15_]^+^ (3/25), 121.1/120.1 [C_8_H_10_N]^+^ (6/64), 92.1/91.1 [Bn]^+^ (9/100)—HRMS (ESI) calcd. [M+Na]^+^ 298.17775; found 298.17775.

*tert-Butyl 4-Azidopentyl(benzyl)carbamate (* ***8*** *).* NaN_3_ (1.586 g, 24.40 mmol) was added to a solution of the bromine derivative** 5** (2.873 g, 8.06 mmol) in DMF (dry, 25 mL) and stirred at 25°C under nitrogen atmosphere for 3 hours. DMF was removed azeotropically with toluene under reduced pressure. The residue was suspended in dichloromethane, filtered through Celite, and concentrated. Purification with flash column chromatography on silica gel (DCM 100%) gave compound** 8** (2.498 g, 97%) as colorless oil. IR (ATR-FTIR): $\tilde{\nu }$ = 2973 (w), 2930 (w), 2359 (w), 2341 (w), 2097 (m), 1688 (s), 1495 (w), 1454 (m), 1413 (m), 1390 (w), 1380 (w), 1364 (m), 1241 (m), 1165 (s), 1138 (s), 1073 (w), 1028 (w), 966 (w), 875 (m), 802 (w), 768 (w), 733 (m), 699 (m), 669 (w), 647 (w), 630 (w), 620 (w)  cm^−1^—^1^H-NMR (600 MHz, CDCl_3_): *δ* = 1.20 (d, ^3^ *J*_H–H_ = 6.54 Hz, 3 H, Me), 1.42–1.60 (m, 13 H,* t*Bu-Me, 2-CH_2_, 3-CH_2_), 3.11–3.20 (m, 2 H, 1-CH_2_), 3.37–3.40 (m, 1 H, 4-H), 4.39–4.42 (m, 2 H, CH_2_Ph), 7.20–7.25 (m, 3 H,* o*-Ph-H,* p*-Ph-H), 7.29–7.31 (m, 2 H,* m*-Ph-H) ppm—^13^C-NMR (150 MHz, CDCl_3_): *δ* = 19.65 (Me), 24.60 (C-2), 24.89 (C-2), 28.63 (*t*Bu-Me), 33.52 (C-3), 46.20 (C-1), 50.04 (CH_2_Ph), 50.57 (CH_2_Ph), 57.85 (C-4), 79.96 (*t*Bu-C), 127.37 (*o*-Ph-CH), 127.92 (*p*-Ph-CH), 128.69 (*m*-Ph-CH), 138.54 (Ph-C), 138.72 (Ph-C), 155.85 (Boc CO), 156.20 (Boc CO) ppm—MS (EI, 70 eV): *m*/*z* (%) = 218.2/217.2 [M-C_5_H_9_O_2_]^+^ (1/7), 92.1/91.1 [C_7_H_7_]^+^ (7/80), 57.1 (100)—HRMS (ESI) calcd. [M+Na]^+^ 341.19480; found 341.19478.

*4-Azido-N-benzylpentan-1-amine (* ***9*** *).* TFA (921.0 mg, 8.08 mmol, and 600 *µ*L) was added to a solution of azide** 8** (51.4 mg, 0.16 mmol) in DCM (1.5 mL) at 25°C and the reaction mixture was stirred for 30 min at 25°C. The mixture was carefully alkalised using aqueous NaOH solution and was exhaustively extracted with dichloromethane. The combined organic extracts were dried (MgSO_4_), filtered, and concentrated. Purification with flash column chromatography on deactivated silica gel (DCM/MeOH 50 : 1) gave** 9** (33.6 mg, 96%) as colorless oil. IR (ATR-FTIR): $\tilde{\nu }$ = 3085 (w), 3062 (w), 3026 (w), 2972 (w), 2931 (w), 2860 (w), 2813 (w), 2358 (w), 2343 (w), 2094 (s), 1603 (w), 1494 (w), 1453 (m), 1379 (w), 1326 (w), 1244 (m), 1188 (w), 1116 (m), 1074 (w), 1027 (w), 968 (w), 908 (w), 805 (w), 731 (s), 697 (s), 670 (w), 653 (w), 622 (w), 610 (w), 600 (w) cm^−1^—^1^H-NMR (600 MHz, CDCl_3_): *δ* = 1.23 (d, ^3^ *J*_H–H_ = 6.60 Hz, 3 H, Me), 1.46–1.57 (m, 3 H, 2-H, 3-CH_2_), 1.58–1.65 (m, 1 H, 2-H), 1.70 (s, br, 1 H, NH), 2.63 (t, ^3^ *J*_H–H_ = 6.90 Hz, 2 H, 1-CH_2_), 3.39–3.45 (m, 1 H, 4-H), 3.77 (s, 2 H, CH_2_Ph), 7.22–7.25 (m, 1 H,* p*-Ph-H), 7.30-7.31 (m, 4 H,* o*-Ph-H,* m*-Ph-H) ppm—^13^C-NMR (150 MHz, CDCl_3_): *δ* = 19.67 (Me), 26.72 (C-2), 34.11 (C-3), 49.07 (C-1), 54.10 (CH_2_Ph), 58.08 (C-4), 127.22 (*p*-Ph-CH), 128.37 (Ph-CH), 128.64 (Ph-CH), 140.26 (Ph-C) ppm—MS (EI, 70 eV): *m*/*z* (%) = 218.1/217.1 [M-H]^+^ (2/16), 92.1/91.1 [C_7_H_7_]^+^ (9/100)—HRMS (ESI) calcd. [M+H]^+^ 219.16042; found 219.16042.

*tert-Butyl 4-Aminopentyl(benzyl)carbamate (* ***10*** *).* NaN_3_ (26.8 mg, 0.41 mmol) and PPh_3_ (102.6 mg, 0.39 mmol) were added to a solution of the bromine derivative** 5** (42.2 mg, 0.12 mmol) in DMF (dry, 1 mL) at 25°C under nitrogen atmosphere. The reaction mixture was stirred for 2.5 hours until the conversion of** 5** to the corresponding amine and iminophosphorane (thin-layer chromatography on silica gel and eluting with petroleum ether/EtOAc 5 : 1, followed by deactivation of the silica gel by gaseous ammonia using DCM/MeOH 10 : 1). For the hydrolysis of the iminophosphorane, KOH (39.3 mg, 0.70 mmol) was added and stirred at 25°C for 16 hours. Water was added and the aqueous phase was exhaustively extracted using dichloromethane. The combined organic extracts were dried (MgSO_4_), filtered, and concentrated. DMF residues were removed using high vacuum. Purification with flash column chromatography on basic alumina (activity level V, DCM/MeOH 30 : 1) gave compound** 10** (25.2 mg, 75%) as colorless oil. IR (ATR-FTIR): $\tilde{\nu }$ = 2971 (w), 2926 (w), 1685 (s), 1604 (w), 1495 (w), 1453 (m), 1413 (m), 1390 (m), 1364 (m), 1241 (m), 1164 (s), 1143 (s), 1091 (m), 1074 (w), 1028 (w), 1002 (w), 966 (w), 882 (m), 812 (w), 769 (w), 731 (m), 699 (m), 672 (w), 638 (w), 620 (w), 606 (w) cm^−1^—^1^H-NMR (600 MHz, CDCl_3_): *δ* = 1.03 (d, ^3^ *J*_H–H_ = 5.76 Hz, 3 H, Me), 1.27 (m, 2H, 3-CH_2_), 1.40–1.47 (m, 11 H,* t*Bu-Me, 2-CH_2_), 2.04 (m, 2 H, NH_2_), 2.84–2.88 (m, 1 H, 4-H), 3.09–3.20 (m, 2 H, 1-CH_2_), 4.38–4.42 (d, ^2^ *J*_H–H_ = 25.80 Hz, 2 H, CH_2_Ph), 7.19–7.24 (m, 3 H,* o*-Ph-CH,* p*-Ph-CH), 7.28–7.30 (m, 2 H,* m*-Ph-CH) ppm—^13^C-NMR (150 MHz, CDCl_3_): *δ* = 23.69 (Me), 24.85 (C-2), 25.20 (C-2), 28.64 (*t*Bu-Me), 36.77 (C-3), 37.01 (C-3), 46.64 (C-1), 46.76 (C-1), 46.94 (C-4), 47.03 (C-4), 50.05 (CH_2_Ph), 50.71 (CH_2_Ph), 79.80 (*t*Bu-C), 79.91 (*t*Bu-C), 127.31 (*o*-Ph-CH), 127.91 (*p*-Ph-CH), 128.66 (*m*-Ph-CH), 138.67 (Ph-C), 138.85 (Ph-C), 155.85 (Boc CO), 156.28 (Boc CO) ppm—MS (EI, 70 eV): *m*/*z* (%) = 293.3/292.3 [M]^+*·*^ (3/12), 237.2/236.2 [M-C_4_H_8_]^+***·***^ (3/18), 192.2/191.2 [M-C_5_H_9_O_2_]^+^ (6/16), 92.1/91.1 [C_7_H_7_]^+^ (10/100)—HRMS (ESI) calcd. [M+H]^+^ 293.22235; found 293.22235.

The intermediary iminophosphorane compound (not displayed) was obtained in experiments without a KOH hydrolysis in various amounts as colorless solid. Mp 63°C (DCM/MeOH)—IR (ATR-FTIR): $\tilde{\nu }$ = 3054 (w), 2975 (w), 2964 (w), 2817 (w), 2717 (w), 2359 (w), 2342 (w), 1682 (s), 1588 (w), 1454 (w), 1436 (m), 1415 (m), 1364 (w), 1310 (w), 1268 (m), 1245 (m), 1222 (w), 1165 (s), 1135 (s), 1112 (s), 1058 (w), 1027 (w), 997 (w), 968 (w), 876 (w), 813 (w), 723 (s), 692 (s), 617 (w) cm^−1^—^1^H-NMR (600 MHz, CDCl_3_): *δ* = 1.28–1.32 (m, br, 6 H, Me), 1.35–1.41 (m, 22 H,* t*Bu-Me, 2-CH_2_), 1.45–1.66 (m, br, 4 H, 3-CH_2_), 2.77–2.88 (m, 2 H, 4-H), 2.91–3.06 (m, 4 H, 1-CH_2_), 4.24–4.39 (m, 4 H, CH_2_Ph), 7.12-7.13 (m, 4 H, Ph-H), 7.18–7.20 (m, 2 H,* p*-Ph-H), 7.23–7.27 (m, 4 H, Ph-H), 7.56–7.59 (m, 12 H, Ph-H), 7.68–7.70 (m, 6 H,* p*-Ph-H), 7.80–7.83 (m, 12 H, Ph-H) ppm—^13^C-NMR (150 MHz, CDCl_3_): *δ* = 22.91 (Me), 22.93 (Me), 25.03 (C-2), 25.64 (C-2), 28.54 (*t*Bu-Me), 28.63 (*t*Bu-Me), 35.26 (C-3), 35.30 (C-3), 35.43 (C-3), 35.47 (C-3), 46.27 (C-1), 46.46 (C-1), 49.93 (CH_2_Ph), 50.68 (C-4), 50.72 (CH_2_Ph), 50.95 (C-4), 79.74 (*t*Bu-C), 79.79 (*t*Bu-C), 122.16 (P-Ph-C), 122.85 (P-Ph-C), 127.16 (Ph-CH), 127.25 (Ph-CH), 127.30 (Ph-CH), 127.71 (Ph-CH), 128.55 (Ph-CH), 128.61 (Ph-CH), 129.83 (P-Ph-CH), 129.92 (P-Ph-CH), 133.83 (P-Ph-CH), 133.91 (P-Ph-CH), 134.64 (P-*p*-Ph-CH), 134.68 (P-*p*-Ph-CH), 138.46 (Ph-C), 138.80 (Ph-C), 155.70 (Boc CO), 156.13 (Boc CO) ppm—^31^P-NMR: (160 MHz, CDCl_3_): 35.94 ppm—MS (EI, 70 eV): *m*/*z* (%) = 537.2 [M-Me]^+***·***^ (4), 362.2/361.2 [M-Bn-Boc]^+***·***^ (1/4), 306.1/305.1/304.1 [Ph_3_PNC_2_H_4_]^+***·***^ (3/24/100), 263.1/262.1 [PPh_3_]^+***·***^ (8/38), 92.1/91.1 [C_7_H_7_]^+^ (2/17)—HRMS (ESI) calcd. [M+H]^+^ 553.29784; found 553.29784.

*tert-Butyl Benzyl(4*′*-(7*′*-chloroquinolin-4*′*-ylamino)pentyl)carbamate (* ***11*** *).* To a solution of 4,7-dichloroquinoline (330.4 mg, 1.67 mmol) in 1,4-dioxane (dry, 8 mL), a 10 min stirred suspension of Pd_2_(dba)_3_ (37.5 mg, 0.04 mmol) and (±)-BINAP (54.7 mg, 0.09 mmol) in 1,4-dioxane (dry, 2 mL) was added at 25°C under nitrogen atmosphere, followed by the amine** 10** (730.2 mg, 2.50 mmol) and KO*t*Bu (373.2 mg, 3.33 mmol). The reaction mixture was stirred at 85°C for 4 hours; after allowing cooling down to 25°C, the mixture was filtered and concentrated. Purification with flash column chromatography on silica gel (petroleum ether/EtOAc 3 : 1) and recrystallization (diethyl ether/petroleum ether) gave** 11** (477.7 mg, 63%) as colorless crystals. Mp 104°C (diethyl ether/petroleum ether)—IR (ATR-FTIR): $\tilde{\nu }$ = 3734 (w), 3628 (w), 3595 (w), 3227 (w), 3064 (w), 3007 (w), 2964 (w), 2920 (w), 2360 (m), 2341 (m), 1679 (s), 1611 (w), 1572 (s), 1549 (m), 1494 (w), 1465 (m), 1451 (m), 1432 (m), 1417 (m), 1395 (w), 1362 (m), 1329 (m), 1315 (m), 1273 (m), 1245 (m), 1214 (m), 1165 (m), 1152 (s), 1133 (s), 1104 (w), 1084 (m), 1067 (w), 1031 (w), 1001 (w), 967 (w), 943 (w), 911 (w), 872 (m), 851 (m), 835 (m), 818 (m), 767 (m), 732 (m), 694 (m), 669 (w), 647 (m), 623 (w), 602 (m) cm^−1^—^1^H-NMR (600 MHz, CDCl_3_): *δ* = 1.25 (d, ^3^ *J*_H–H_ = 6.36 Hz, 3 H, Me), 1.44 (s, 9 H,* t*Bu-Me), 1.50–1.64 (m, 4 H, 2-CH_2_, 3-CH_2_), 3.14 (m, br, 1 H, 1-CH_2_), 3.41–3.45 (m, br, 1 H, 1-CH_2_), 3.68 (m, br, 1 H, 4-H), 4.30–4.43 (m, 2 H, CH_2_Ph), 5.71 (s, br, NH), 6.35 (m, br, 1 H, 3′-H), 7.17–7.28 (m, 5 H,* o*-Ph-H,* m*-Ph-H,* p*-Ph-H), 7.32 (dd, ^3^ *J*_H–H_ = 8.88 Hz, ^4^ *J*_H–H_ = 1.92 Hz, 1 H, 6′-H), 7.87 (m, br, 1 H, 5′-H), 7.94 (d, ^4^ *J*_H–H_ = 1.50 Hz, 1 H, 8′-H), 8.45 (m, br, 1 H, 2′-H) ppm—^13^C-NMR (150 MHz, CDCl_3_): *δ* = 20.74 (Me), 25.06 (CH_2_), 25.25 (CH_2_), 28.63 (*t*Bu-Me), 32.83 (CH_2_), 46.48 (C-1), 48.21 (C-4), 49.24 (C-4), 50.79 (CH_2_Ph), 80.00 (*t*BuC), 80.38 (*t*Bu-C), 99.15 (C-3′), 117.45 (C-4′a), 122.06 (C-5′), 125.43 (C-6′), 127.23 (*o*-Ph-C), 127.46 (*p*-Ph-C), 127.93 (C-8′), 128.25 (C-8′), 128.71 (*m*-Ph-C), 135.29 (C-7′), 138.53 (Ph-C), 148.78 (C-8′a), 148.98 (C-8′a), 149.82 (C-4′), 151.38 (C-2′), 152.01 (C-2′), 156.35 (Boc CO) ppm—MS (EI, 70 eV): *m*/*z* (%) = 455.2/454.2/453.2 [M]^+***·***^ (19/17/49), 398.1/397.1/396.1 [M-C_4_H_9_]^+^ (6/7/13), 221.0/220.0/219.0 [C_12_H_12_ClN_2_]^+^ (11/7/34), 207.0/206.0/205.0 [C_11_H_10_ClN_2_]^+^ (35/26/100), 92.0/91.0 [C_7_H_7_]^+^ (9/91)—HRMS (ESI) calcd. [M+H]^+^ 454.22558; found 454.22559.

*N* ^*1*^ *-Benzyl-N* ^*4*^ *-(7*′*-chloroquinolin-4*′*-yl)pentane-1,4-diamine (* ***12*** *).* TFA (1.432 g, 12.56 mmol, and 933 *µ*L) was added to a solution of the Boc protected** 11** (114.1 mg, 0.25 mmol) in DCM (15 mL) and the reaction mixture was stirred at 25°C for 1.5 hours. The mixture was carefully alkalised with aqueous NaOH solution and was exhaustively extracted with dichloromethane. The combined organic extracts were dried (MgSO_4_), filtered, and concentrated. Purification with flash column chromatography on deactivated silica gel (DCM/MeOH 30 : 1) and recrystallization (DCM/diethyl ether/petroleum ether) gave** 12** (77.5 mg, 88%) as light beige solid. Mp 88°C (DCM/diethyl ether/petroleum ether)—IR (ATR-FTIR): $\tilde{\nu }$ = 3247 (w), 3062 (w), 3027 (w), 2958 (w), 2861 (w), 2815 (w), 2790 (w), 2360 (w), 2337 (w), 1610 (w), 1569 (s), 1542 (m), 1490 (w), 1452 (m), 1425 (m), 1369 (m), 1328 (m), 1280 (w), 1251 (m), 1201 (w), 1137 (m), 1106 (m), 1078 (m), 1041 (w), 1008 (w), 956 (w), 902 (m), 867 (m), 848 (m), 817 (m), 792 (m), 738 (m), 698 (s), 638 (m) cm^−1^—^1^H-NMR (600 MHz, MeOD-d_4_): *δ* = 1.30 (d, ^3^ *J*_H–H_ = 6.42 Hz, 3 H, Me), 1.60–1.66 (m, 3 H, 2-CH_2_, 3-H), 1.70–1.75 (m, 2 H, 3-CH_2_), 2.59 (t, ^3^ *J*_H–H_ = 7.08 Hz, 2 H, 1-CH_2_), 3.70 (s, 2 H, CH_2_Ph), 3.76–3.78 (m, 1 H, 4-H), 6.50 (d, ^3^ *J*_H–H_ = 5.76 Hz, 1 H, 3′-H), 7.20–7.23 (m, 1 H,* p*-Ph-H), 7.27-7.28 (m, 4 H,* o*-Ph-H,* m*-Ph-H), 7.37 (dd, ^3^ *J*_H–H_ = 9.03 Hz, ^4^ *J*_H–H_ = 2.19, 1 H, 6′-H), 7.76 (d, ^4^ *J*_H–H_ = 2.10 Hz, 1 H, 8′-H), 8.16 (d, ^3^ *J*_H–H_ = 9.00 Hz, 1 H, 5′-H), 8.31 (d, ^3^ *J*_H–H_ = 5.64 Hz, 2′-H) ppm—^13^C-NMR (150 MHz, MeOD-d_4_): *δ* = 20.50 (Me), 27.30 (C-2), 35.02 (C-3), 49.58 (C-4), 49.78 (C-1), 54.55 (CH_2_Ph), 99.97 (C-3′), 118.98 (C-4′a), 124.62 (C-5′), 125.96 (C-6′), 127.71 (C-8′), 128.30 (*p*-Ph-CH), 129.58 (Ph-CH), 129.66 (Ph-CH), 136.43 (C-7′), 140.71 (Ph-C), 150.04 (C-8′a), 152.28 (C-2′), 152.58 (C-4′) ppm—MS (EI, 70 eV): *m*/*z* (%) = 354.2/353.2 [M]^+***·***^ (6/12), 264.1/263.1/262.1 [M-Bn]^+^ (10/7/29), 234.0/233.0 [C_13_H_14_ClN_2_]^+^ (7/9), 221.1/220.1/219.1 [C_11_H_12_ClN_2_]^+^ (7/5/22), 207.0/206.0/205.0 [C_11_H_10_ClN_2_]^+^ (13/20/33), 178.0 [C_9_H_7_ClN_2_]^+^ (12), 91.1 [Bn]^+^ (100)—HRMS (ESI) calcd. 354.17315 [M+H]^+^; found 354.17307.

*tert-Butyl 4-(7*′*-Chloroquinolin-4*′*-ylamino)butylcarbamate (* ***14*** *).* Boc_2_O (199.5 mg, 0.91 mmol, and 210 *µ*L) was added to a solution of** 13 **[37] (203.0 mg, 0.81 mmol) in MeCN (dry, 6 mL) at 0°C under nitrogen atmosphere, and the reaction mixture was stirred for 1 hour at 25°C and concentrated. The residue was suspended in water and alkalized with aqueous NaOH solution and the mixture was exhaustively extracted with dichloromethane. The combined organic extracts were dried (MgSO_4_), filtered, and concentrated. Purification with flash column chromatography on deactivated silica gel (DCM/MeOH 40 : 1) and recrystallization (CHCl_3_/petroleum ether) yielded in compound** 14 **(258.9 mg, 91%) as beige crystals. Mp 176°C (CHCl_3_/petroleum ether)—IR (ATR-FTIR): $\tilde{\nu }$ = 3344 (w), 3181 (w), 3003 (w), 2982 (w), 2961 (w), 2930 (w), 2864 (w), 2360 (w), 2341 (w), 1677 (s), 1610 (w), 1578 (s), 1541 (s), 1487 (w), 1468 (w), 1449 (m), 1427 (m), 1389 (w), 1365 (m), 1354 (m), 1330 (m), 1306 (w), 1286 (s), 1274 (s), 1249 (m), 1234 (m), 1162 (s), 1141 (s), 1124 (s), 1081 (w), 1036 (w), 1021 (w), 994 (m), 951 (m), 895 (w), 884 (m), 852 (m), 828 (w), 818 (s), 770 (m), 751 (m), 715 (w), 668 (w), 643 (m), 624 (w) cm^−1^—^1^H-NMR (600 MHz, MeOD-d_4_): *δ* = 1.42 (s, 9 H,* t*Bu-Me), 1.59–1.64 (m, 2 H, 2-CH_2_), 1.73–1.78 (m, 2 H, 3-CH_2_), 3.11 (t, ^3^ *J*_H–H_ = 6.90 Hz, 2 H, 1-CH_2_), 3.38 (t, ^3^ *J*_H–H_ = 7.11 Hz, 2 H, 4-CH_2_), 6.53 (d, ^3^ *J*_H–H_ = 5.76 Hz, 1 H, 3′-H), 7.39 (dd, ^3^ *J*_H–H_ = 8.97 Hz, ^4^ *J*_H–H_ = 2.13 Hz, 1 H, 6′-H), 7.77 (d, ^4^ *J*_H–H_ = 1.98 Hz, 1 H, 8′-H), 8.11 (d, ^3^ *J*_H–H_ = 8.94 Hz, 1 H, 5′-H), 8.34 (d, ^3^ *J*_H–H_ = 5.64 Hz, 1 H, 2′-H) ppm—^13^C-NMR (150 MHz, MeOD-d_4_): *δ* = 26.66 (C-3), 28.78 (C-2), 28.92 (*t*Bu-Me), 41.10 (C-1), 43.81 (C-4), 80.06 (*t*Bu-C), 99.79 (C-3′), 118.91 (C-4′a), 124.54 (C-5′), 126.10 (C-6′), 127.60 (C-8′), 136.49 (C-7′), 149.72 (C-8′a), 152.45 (C-2′), 152.95 (C-4′), 158.81 (Boc CO) ppm—MS (EI, 70 eV): *m*/*z* (%) = 351.0/350.1/349.1 [M]^+***·***^ (12/8/33), 295.0/294.0/293.0 [M-C_4_H_8_]^+***·***^ (7/6/22), 251.0/250.0/249.0 [M-C_5_H_8_O_2_]^+***·***^ (2/2/6), 193.0/192.0/191.0 [C_10_H_8_ClN_2_]^+^ (34/21/100), 180.0/179.0/178.0 [C_9_H_7_ClN_2_]^+***·***^ (10/32/19)—HRMS (ESI) calcd. [M+H]^+^ 350.16298; found 350.16297.

*tert-Butyl Benzyl(4-(benzyl(7*′*-chloroquinolin-4*′*-yl)amino)butyl)carbamate (* ***15*** *).* NaH (51.4 mg of a 55% oily dispersion, 1.18 mmol) was added to a solution of the Boc protected** 14** (134.8 mg, 0.39 mmol) in DMF (dry, 2 mL) under nitrogen atmosphere at 0°C and the reaction mixture was stirred for 30 min. Benzyl bromide (50.4 *µ*L, 72.5 mg, and 0.42 mmol) was added at 0°C, and the mixture was allowed to warm up to 25°C and was stirred for further 6 hours. DMF was removed using high vacuum and the residue was purified with flash column chromatography on silica gel (petroleum ether/EtOAc 2 : 1) to give compounds** 15** (47.2 mg, 23%) and** 16** (9.1 mg, 23%) both as highly viscous colorless oil. Compound** 15**: IR (ATR-FTIR): $\tilde{\nu }$ = 3062 (w), 3027 (w), 2971 (w), 2931 (w), 2861 (w), 2360 (w), 1687 (s), 1604 (w), 1565 (s), 1494 (m), 1454 (m), 1417 (s), 1363 (s), 1295 (m), 1240 (m), 1162 (s), 1139 (s), 1074 (m), 1051 (w), 1029 (w) cm^−1^—^1^H-NMR (600 MHz, MeOD-d_4_): *δ* = 1.36 (s, 9 H,* t*Bu-Me), 1.41–1.43 (m, 2 H, 2-CH_2_), 1.51–1.55 (m, 2 H, 3-CH_2_), 3.08–3.15 (m, 2 H, 1-CH_2_), 3.25 (m, 2 H, 4-CH_2_), 4.28–4.31 (m, 2 H, CH_2_Ph), 4.51 (s, br, 2 H, CH_2_Ph), 6.90 (d, ^3^ *J*_H–H_ = 5.28 Hz, 1 H, 3′-H), 7.11-7.12 (m, 2 H, Ph-CH), 7.16–7.19 (m, 1 H, Ph-CH), 7.21–7.29 (m, 7 H, Ph-CH), 7.44 (d, ^3^ *J*_H–H_ = 8.58 Hz, 1 H, 6′-H), 7.92 (s, 1 H, 8′-H), 8.11 (d, ^3^ *J*_H–H_ = 8.94 Hz, 1 H, 5′-H), 8.51 (s, br, 1 H, 2′-H) ppm—^13^C-NMR (150 MHz, MeOD-d_4_): *δ* = 25.13 (C-3), 25.25 (C-3), 26.62 (C-2), 27.15 (C-2), 28.80 (*t*Bu-Me), 47.92 (C-1), 51.30 (CH_2_Ph), 51.89 (CH_2_Ph), 53.00 (C-4), 53.26 (C-4), 58.35 (CH_2_Ph), 58.73 (CH_2_Ph), 123.91 (C-4′a), 127.17 (C-6′), 127.41 (C-5′), 128.29 (Ph-CH), 128.39 (Ph-CH), 128.52 (C-8′), 129.10 (Ph-CH), 129.64 (Ph-CH), 129.79 (Ph-CH), 136.58 (C-7′), 138.75 (Ph-C), 139.76 (Ph-C), 140.02 (Ph-C), 151.14 (C-8′a), 152.22 (C-2′), 157.53 (Boc CO), 157.80 (Boc CO), 158.06 (C-4′) ppm—MS (EI, 70 eV): *m*/*z* (%) = 531.0/530.0/529.0 [M]^+***·***^ (8/8/18), 474.0/473.0/472.0 [M-C_4_H_9_]^+^ (3/2/6), 440.0/439.0/438.0 [M-Bn]^+^ (6/5/16), 91 [Bn]^+^ (100)—HRMS (ESI) calcd. [M+H]^+^ 530.25688; found 530.25689.

*tert-Butyl 4-(Benzyl(7*′*-chloroquinolin-4*′*-yl)amino)butylcarbamate (* ***16*** *).* IR (ATR-FTIR): $\tilde{\nu }$ = 3241 (w, br), 3031 (w), 2973 (w), 2931 (w), 2863 (w), 1693 (s), 1604 (m), 1565 (s), 1496 (s), 1452 (m), 1425 (s), 1388 (m), 1363 (s), 1292 (s), 1272 (s), 1247 (s), 1164 (s), 1076 (m), 1045 (m), 997 (m) cm^−1^—^1^H-NMR (600 MHz, MeOD-d_4_): *δ* = 1.38 (s, 9 H,* t*Bu-Me), 1.40–1.45 (m, 2 H, 2-CH_2_), 1.65–1.70 (m, 2 H, 3-CH_2_), 2.98 (t, ^3^ *J*_H–H_ = 6.75 Hz, 2 H, 1-CH_2_), 3.31–3.34 (m, 2 H, 4-CH_2_), 4.58 (s, 2 H, CH_2_Ph), 6.95 (d, ^3^ *J*_H–H_ = 5.34 Hz, 1 H, 3′-H), 7.22–7.24 (m, 1 H,* p*-Ph-H), 7.27–7.30 (m, 4 H,* o*-Ph-H,* m*-Ph-H), 7.46 (dd, ^3^ *J*_H–H_ = 9.06 Hz, ^4^ *J*_H–H_ = 1.98 Hz, 1 H, 6′-H), 7.91 (d, ^4^ *J*_H–H_ = 1.92 Hz, 1 H, 8′-H), 8.16 (d, ^3^ *J*_H–H_ = 9.06 Hz, 1 H, 5′-H), 8.51 (d, ^3^ *J*_H–H_ = 5.22 Hz, 1 H, 2′-H) ppm—^13^C-NMR (150 MHz, MeOD-d_4_): *δ* = 25.18 (C-3), 28.60 (C-2), 28.92 (*t*Bu-Me), 41.05 (C-1), 53.43 (C-4), 58.34 (CH_2_Ph), 79.98 (*t*Bu-C), 112.19 (C-3′), 123.87 (C-4′a), 127.09 (C-6′), 127.48 (C-5′), 128.44 (C-8′), 128.66 (*p*-Ph-CH), 129.07 (*o*-Ph-CH), 129.77 (*m*-Ph-CH), 136.55 (C-7′), 138.80 (Ph-C), 151.13 (C-8′a), 152.19 (C-2′), 158.15 (C-4′), 158.67 (Boc CO) ppm—MS (EI, 70 eV): *m*/*z* (%) = 441.0/440.0/439.0 [M]^+***·***^ (3/3/7), 91 [Bn]^+^ (100)—HRMS (ESI) calcd. [M+H]^+^ 440.20993; found 440.20993.

*tert-Butyl 4-(6*′*-Methoxyquinolin-8*′*-ylamino)pentylcarbamate (* ***27*** *).* Boc_2_O (2.755 g, 12.6 mmol, and 2.9 mL) was added to a solution of the free base of the commercially available primaquine biphosphate (1.605 g, 6.19 mmol) in DCM (dry, 40 mL) at 0°C and the reaction mixture was stirred for 1 hour at 25°C. The organic layer was washed with aqueous NaHCO_3_ solution, dried (MgSO_4_), and concentrated. Purification with flash column chromatography on deactivated silica gel (petroleum ether/EtOAc 5 : 1) gave compound** 27** (2.081, 94%) as yellow oil. IR (ATR-FTIR): $\tilde{\nu }$ = 3380 (w, br), 2965 (w), 2931 (w), 2867 (w), 2501 (w), 2360 (w), 2339 (w), 1691 (s), 1612 (m), 1577 (m), 1515 (s), 1452 (m), 1405 (s), 1386 (s), 1365 (m), 1338 (m), 1245 (m), 1209 (s), 1160 (s), 1074 (s), 1035 (m), (970), 865 (w), 815 (m), 786 (m), 663 (m), 622 (m) cm^−1^—^1^H-NMR (600 MHz, MeOD-d_4_): *δ* = 1.25 (d, ^3^ *J*_H–H_ = 6.36 Hz, 3 H, Me), 1.40 (s, 9 H,* t*Bu-Me), 1.54–1.68 (m, 4 H, 2-H, 3-H), 3.02–3.06 (m, 2 H, 1-H), 3.59–3.62 (m, 1 H, 4-H), 3.85 (s, 3 H, OMe), 6.30 (s, 1 H, 7′-H), 6.42 (d, ^4^ *J*_H–H_ = 2.40 Hz, 1 H, 5′-H), 7.32 (dd, ^3^ *J*_H–H_ = 4.20 Hz, 8.22 Hz, 1 H, 3′-H), 7.99 (d, ^3^ *J*_H–H_ = 8.28 Hz, ^4^ *J*_H–H_ = 1.56 Hz, 1 H, 4′-H), 8.47 (dd, ^3^ *J*_H–H_ = 4.20 Hz, ^4^ *J*_H–H_ = 1.56 Hz, 1 H, 2′-H) ppm—^13^C-NMR (150 MHz, MeOD-d_4_): *δ* = 20.86 (Me), 27.74 (C-2), 28.91 (*t*Bu-Me), 35.00 (C-3), 41.47 (C-1), 49.10 (C-4), 55.77 (OMe), 79.95 (*t*Bu-C), 93.18 (C-5′), 98.59 (C-7′), 123.01 (C-3′), 131.65 (C-4′a), 136.43 (C-4′), 136.56 (C-8′a), 145.40 (C-2′), 146.22 (C-8′), 158.66 (Boc-CO), 161.06 (C-6′) ppm—MS (EI, 70 eV): *m*/*z* (%) = 361.2/360.2/359.2 [M]^+***·***^ (3/9/9), 260.2/259.2 [M-C_5_H_8_O_2_]^+***·***^ (5/5), 203.1/202.1/201.1 [C_12_H_13_N_2_O]^+^ (16/68/100)—HRMS (ESI) calcd. [M+Na]^+^ 382.21011; found 382.21011.

*tert-Butyl Benzyl(4-(6*′*-methoxyquinolin-8*′*-ylamino)pentyl)carbamate (* ***28*** *).* NaH (23.6 mg of a 55% oily dispersion, 0.25 mmol) was added at 0°C under nitrogen atmosphere to a solution of Boc protected** 27** (69.5 mg, 0.19 mmol) in DMF (dry, 2 mL) and the reaction mixture was stirred for 30 min at 0°C. At 25°C benzyl bromide (25.3 *µ*L, 36.4 mg, and 0.22 mmol) was added and the mixture was stirred for further 1.5 hours. DMF was removed using high vacuum and the residue was purified with flash column chromatography on silica gel (petroleum ether/EtOAc 8 : 1) to give compound** 28** (67.5 mg, 79%) as yellow oil. IR (ATR-FTIR): $\tilde{\nu }$ = 3384 (w, br), 2965 (w), 2929 (w), 2360 (w), 2337 (w), 2086 (w, br), 1862 (w, br), 1687 (s), 1614 (m), 1575 (m), 1517 (s), 1454 (s), 1415 (s), 1386 (s), 1365 (m), 1241 (m), 1213 (m), 1159 (s), 1087 (w), 1051 (m), 1029 (w) cm^−1^—^1^H-NMR (600 MHz, MeOD-d_4_): *δ* = 1.23-1.24 (m, 6 H, Me), 1.39 (d, ^4^ *J*_H–H_ = 16.26 Hz, 18 H,* t*Bu-Me), 1.56–1.58 (m, 4 H, CH_2_), 1.59–1.62 (m, 4 H, CH_2_), 3.14-3.15 (m, 2 H, 1-H), 3.19–3.28 (m, 2 H, 1-H), 3.58–3.60 (m, 2 H, 4-H), 3.86 (s, 6 H, OMe), 4.29–4.40 (m, 4 H, CH_2_Ph), 6.28 (d, ^3^ *J*_H–H_ = 12.78 Hz, 2 H, 7′-H), 6.46 (d, ^3^ *J*_H–H_ = 2.40 Hz, 2 H, 5′-H), 7.13–7.20 (m, 6 H, Ph-H), 7.24–7.26 (m, 4 H, Ph-H), 7.37 (dd, ^3^ *J*_H–H_ = 4.23 Hz, 8.19 Hz, 2 H, 3′-H), 8.04 (dd, ^3^ *J*_H–H_ = 8.28 Hz, ^4^ *J*_H–H_ = 1.44 Hz, 2 H, 4′-H), 8.49 (dd, ^3^ *J*_H–H_ = 4.20 Hz, ^4^ *J*_H–H_ = 1.56 Hz, 2 H, 2′-H) ppm—^13^C-NMR (150 MHz, MeOD-d_4_): *δ* = 20.90 (Me), 20.99 (Me), 25.45 (CH_2_), 25.78 (CH_2_), 28.74 (*t*Bu-Me), 34.56 (CH_2_), 34.65 (CH_2_), 47.85 (C-1), 48.07 (C-1), 48.70 (C-4), 51.06 (CH_2_Ph), 51.55 (CH_2_Ph), 55.75 (OMe), 81.30 (*t*Bu-C), 81.38 (*t*Bu-C), 93.01 (C-5′), 98.61 (C-7′), 123.12 (C-3′), 128.34 (Ph-CH), 128.39 (Ph-CH), 128.44 (Ph-CH), 128.77 (Ph-CH), 129.64 (Ph-CH), 129.70 (Ph-CH), 131.79 (C-4′a), 136.53 (C-4′), 136.57 (C-8′a), 139.68 (Ph-C), 139.98 (Ph-C), 145.45 (C-2′), 146.22 (C-8′), 157.78 (Boc CO), 157.94 (Boc CO), 161.11 (C-6′) ppm—MS (EI, 70 eV): *m*/*z* (%) = 451.3/450.3/449.3 [M]^+***·***^ (2/11/35), 203.1/202.1/201.1 [C_12_H_13_N_2_O]^+^ (1/15/100), 174.1 [C_10_H_10_N_2_O]^+***·***^ (29), 160.1 [C_9_H_8_N_2_O]^+***·***^ (10), 92.1/91.1 [C_7_H_7_]^+^ (2/17)—HRMS (ESI) calcd. [M+H]^+^ 450.27512; found 450.27512.

*tert-Butyl Benzyl(4-(1*′*,3*′*-dioxoisoindolin-2*′*-yl)pentyl)carbamate (* ***30*** *).* Potassium phthalimide (180.0 mg, 1.03 mmol) was added to a solution of the bromine derivative** 5** (168.5 mg, 0.47 mmol) in DMF (dry, 5 mL) and the reaction mixture was stirred at 25°C for 72 hours. DMF was removed azeotropically with toluene under reduced pressure, and the residue was suspended in aqueous NaHCO_3_ solution and exhaustively extracted with dichloromethane. Purification with flash column chromatography on silica gel (petroleum ether/EtOAc 10 : 1) gave compound** 30** (161.5 mg, 81%) as viscous colorless oil. IR (ATR-FTIR): $\tilde{\nu }$ = 2973 (w), 2931 (w), 1773 (w), 1703 (s), 1687 (s), 1612 (w), 1495 (w), 1466 (w), 1453 (m), 1413 (m), 1391 (m), 1363 (s), 1332 (m), 1288 (w), 1243 (m), 1168 (m), 1145 (s), 1088 (w), 1046 (m), 967 (w) cm^−1^—^1^H-NMR (600 MHz, CDCl_3_): *δ* = 1.39–1.42 (m, 24 H,* t*Bu-Me, Me), 1.60–1.68 (m, 6 H, 2-CH_2_, 3-CH_2_), 1.94–1.99 (m, 2 H, 2-CH_2_, 3-CH_2_), 3.02–3.12 (m, 2 H, 1-CH_2_), 3.18 (t, ^3^ *J*_H–H_ = 7.26 Hz, 2 H, 1-CH_2_), 4.27–4.31 (m, 4 H, CH_2_Ph), 4.35 (d, ^2^ *J*_H–H_ = 15.72 Hz, 1 H, CH_2_Ph), 4.44 (d, ^2^ *J*_H–H_ = 15.24 Hz, 1 H, CH_2_Ph), 7.12–7.18 (m, 6 H,* o*-Ph-H,* p*-Ph-H), 7.21–7.23 (m, 4 H,* m*-Ph-H), 7.68–7.71 (m, 4 H, 5′-H, 6′-H), 7.78–7.81 (m, 4 H, 4′-H, 7′-H) ppm—^13^C-NMR (150 MHz, CDCl_3_): *δ* = 18.90 (Me), 18.94 (Me), 25.37 (CH_2_), 25.56 (CH_2_), 28.57 (*t*Bu-Me), 30.98 (CH_2_), 31.06 (CH_2_), 46.10 (1-CH_2_), 46.33 (1-CH_2_), 47.13 (C-4), 47.32 (C-4), 49.96 (CH_2_Ph), 50.65 (CH_2_Ph), 79.84 (*t*Bu-C), 79.91 (*t*Bu-C), 123.29 (C-4′), 127.21 (*o*-Ph-CH), 127.28 (*o*-Ph-CH), 127.86 (*p*-Ph-CH), 128.55 (*m*-Ph-CH), 128.60 (*m*-Ph-CH), 131.96 (C-3′a, C-7′a), 132.03 (C-3′a, C-7′a), 134.05 (C-5′, C-6′), 134.14 (C-5′, C-6′), 138.45 (Ph-C), 138.72 (Ph-C), 168.71 (C-1′, C-3′) ppm—MS (EI, 70 eV): *m*/*z* (%) = 323.2/322.2/321.2 [M-C_5_H_9_O_2_]^+^ (6/22/39), 232.1/231.1 [M-C_7_H_7_-C_5_H_9_O_2_+H]^+^ (2/14), 175.0/174.0 [C_10_H_8_NO_2_]^+^ (6/28), 121.0/120.0 [C_8_H_10_N]^+^ (7/74), 107.0/106.0 [C_7_H_8_N]^+^ (10/65), 92.0/91.0 [C_7_H_7_]^+^ (9/100)—HRMS (ESI) calcd. [M+H]^+^ 323.17540; found 323.17540.

*tert-Butyl 4-(1H-Imidazole-1-yl)pentyl(benzyl)carbamate (* ***29*** *).* NaH (60.7 mg of a 55% oily dispersion, 1.39 mmol) was added to a solution of imidazole (47.3 mg, 0.69 mmol) in DMF (dry, 4 mL) at 0°C under nitrogen atmosphere and the reaction mixture was stirred for 30 min. The bromine compound** 5** (312.2 mg, 0.88 mmol) was added at 0°C, and the reaction mixture was allowed to warm up to 25°C and was stirred for further 7 hours. The excess of NaH was carefully hydrolyzed using a small amount of water and the solvent mixture was removed azeotropically with toluene under reduced pressure. Purification with flash column chromatography on silica gel (EtOAc 100%) gave compound** 29** (118.9 mg, 50%) as viscous colorless oil. IR (ATR-FTIR): $\tilde{\nu }$ = 2972 (w), 2931 (w), 1682 (s), 1495 (w), 1453 (m), 1413 (m), 1391 (w), 1364 (m), 1278 (w), 1241 (m), 1225 (m), 1163 (s), 1138 (s), 1111 (w), 1076 (w), 1027 (w), 1001 (w), 966 (w), 905 (w), 872 (w), 812 (w), 767 (w), 733 (m), 700 (m), 665 (m), 642 (w), 627 (w) cm^−1^—^1^H-NMR (600 MHz, CDCl_3_, +3°C): *δ* = 1.21–1.44 (m, 28 H, Me,* t*Bu-Me, 3-CH_2_,** A**,** B**), 1.61–1.66 (m, 4 H, 2-CH_2_,** A**,** B**), 3.02–3.22 (m, 4 H, 1-CH_2_,** A**,** B**), 4.03–4.09 (m, 1 H, 4-H,** B**), 4.17–4.21 (m, 1 H, 4-H,** A**), 4.30 (s, 2 H, CH_2_Ph,** B**), 4.32 (d, ^2^ *J*_H–H_ = 15.20 Hz, 1 H, CH_2_Ph,** A**), 4.41 (d, ^2^ *J*_H–H_ = 15.24 Hz, 1 H, CH_2_Ph,** A**), 6.87 (s, 1 H, 2′-H,** B**), 6.91 (s, 1 H, 2′-H,** A**), 7.06–7.09 (m, 2 H, 3′-H,** A**,** B**), 7.13–7.18 (m, 4 H, Ph-H,** A**,** B**), 7.23–7.25 (m, 3 H, Ph-H,** A**,** B**), 7.29 (m, 3 H, Ph-H,** A**,** B**), 7.57 (s, 1 H, 5′-H,** B**), 7.66 (s, 1 H, 5′-H,** A**) ppm—^13^C-NMR (150 MHz, CDCl_3_, +3°C): *δ* = 22.23 (Me,** A**), 22.35 (Me,** B**), 24.39 (C-3,** A**), 24.87 (C-3,** B**), 28.55 (*t*Bu-Me,** A**), 28.62 (*t*Bu-Me,** B**), 34.81 (C-2,** A**), 35.07 (C-2,** B**), 45.45 (C-1,** A**), 45.88 (C-1,** B**), 49.95 (CH_2_Ph,** B**), 50.36 (CH_2_Ph,** A**), 53.87 (C-4,** A**,** B**), 80.05 (*t*Bu-C,** B**), 80.19 (*t*Bu-C,** A**), 116.58 (C-2′,** B**), 116.89 (C-2′,** A**), 127.28 (Ph-CH,** A**,** B**), 127.43 (Ph-CH,** A**,** B**), 127.47 (Ph-CH,** A**,** B**), 127.86 (Ph-CH,** A**,** B**), 128.20 (C-3′,** A**), 128.70 (Ph-CH,** A**,** B**), 128.92 (C-3′,** B**), 135.64 (C-5′,** A**), 135.77 (C-5′,** B**), 138.30 (Ph-C,** B**), 138.41 (Ph-C,** A**), 155.92 (Boc CO,** A**), 156.11 (Boc CO,** B**) ppm—MS (EI, 70 eV): *m*/*z* (%) = 344.3/343.3 [M]^+***·***^ (11/37), 287.2/286.2 [M-C_4_H_9_]^+^ (6/5), 271.2/270.2 [M-C_4_H_9_O]^+^ (4/19), 243.2/242.2 [M-C_5_H_9_O_2_]^+^ (3/9), 92.0/91.0 [C_7_H_7_]^+^ (9/100)—HRMS (ESI) calcd. [M+H]^+^ 344.23325; found 344.23325.

*1-Benzyl-2-methylpyrrolidine (* ***17*** *).* TFA (985 *µ*L, 1.512 g, and 13.26 mmol) was added to a solution of bromine derivative** 5** (94.4 mg, 0.26 mmol) in DCM (5 mL) and the reaction mixture was stirred at 25°C for 1 hour. Dichloromethane was added and the organic layer was washed using aqueous NaOH solution. The organic layer was dried (MgSO_4_), filtered, and concentrated. Compound** 17** (43.6 mg, 96%) was obtained as beige crystals. Mp 145°C (DCM)—IR (ATR-FTIR): $\tilde{\nu }$ = 2965 (w), 2919 (w), 2846 (w), 2739 (w), 2654 (m), 2592 (m), 2508 (w), 2480 (w), 2360 (s), 2341 (m), 1683 (w), 1496 (w), 1458 (w), 1440 (w), 1416 (w), 1390 (w), 1353 (w), 1330 (w), 1291 (w), 1148 (w), 1072 (m), 1029 (m), 1000 (w) cm^−1^—^1^H-NMR (600 MHz, CDCl_3_): *δ* = 1.56 (d, ^3^ *J*_H–H_ = 6.42 Hz, 6 H, Me), 1.85–1.92 (m, 2 H, 4-H), 2.01–2.08 (m, 2 H, 3-H), 2.14–2.23 (m, 4 H, 3-H, 4-H), 2.84–2.90 (m, 2 H, 5-H), 3.31–3.38 (m, 2 H, 2-H), 3.61–3.66 (m, 2 H, 5-H), 4.11 (d, ^2^ *J*_H–H_ = 13.32 Hz, 1 H, CH_2_Ph), 4.13 (d, ^2^ *J*_H–H_ = 13.32 Hz, 1 H, CH_2_Ph), 4.29 (d, ^2^ *J*_H–H_ = 13.32 Hz, 1 H, CH_2_Ph), 4.32 (d, ^2^ *J*_H–H_ = 13.32 Hz, 1 H, CH_2_Ph), 7.38–7.42 (m, 6 H, Ph-CH), 7.58–7.60 (m, 4 H, Ph-CH) ppm—^13^C-NMR (150 MHz, CDCl_3_): *δ* = 16.07 (Me), 21.09 (C-4), 31.58 (C-3), 52.85 (C-5), 55.89 (CH_2_Ph), 63.17 (C-2), 128.83 (Ph-C), 129.51 (Ph-CH), 130.26 (Ph-CH), 131.44 (Ph-CH) ppm—MS (EI, 70 eV): *m*/*z* (%) = 176.2/175.2 [M]^+***·***^ (1/10), 161.1/160.1 [M-CH_3_]^+^ (12/100), 92.1/91.1 [C_7_H_7_]^+^ (6/66)—HRMS (ESI) calcd. [M+H]^+^ 176.14338; found 176.14338.

*1,1-Dibenzyl-2-methylpyrrolidinium Bromide (* ***18*** *).* Cs_2_CO_3_ (859.6 mg, 2.64 mmol) was added to a solution of (*rac*)-1,4-dibromopentane (500 *µ*L, 843.5 mg, and 3.67 mmol) and dibenzylamine (300 *µ*L, 307.8 mg, and 1.56 mmol) in acetone (10 mL) and the reaction mixture was stirred for 7 hours at 70°C. The mixture was concentrated, the residue was suspended in aqueous NaHCO_3_ solution, and the aqueous layer was exhaustively extracted with dichloromethane. The combined organic extracts were dried (MgSO_4_), filtered, and concentrated. Purification with flash column chromatography on silica gel (petroleum ether/EtOAc 50 : 1) gave compound** 18** (289.2 mg, 54%) as colorless foam. Mp 198°C (petroleum ether/EtOAc)—IR (ATR-FTIR): $\tilde{\nu }$ = 3726 (w), 3700 (w), 3626 (w), 3595 (w), 3034 (w), 2977 (w), 2921 (w), 2890 (w), 2360 (s), 2341 (s), 1497 (w), 1451 (w), 1406 (w), 1375 (w), 1348 (w), 1311 (w), 1259 (w), 1212 (w), 1186 (w), 1153 (w), 1069 (w), 1036 (w), 1008 (w) cm^−1^—^1^H-NMR (600 MHz, CDCl_3_): *δ* = 1.83 (d, ^3^ *J*_H–H_ = 6.48 Hz, 3 H, Me), 1.94–1.98 (m, 1 H, 4-H), 2.18–2.26 (m, 2 H, 3-CH_2_), 2.28–2.31 (m, 1 H, 4-H), 3.03–3.08 (m, 1 H, 5-H), 3.38–3.41 (m, 1 H, 5-H), 3.52–3.59 (m, 1 H, 2-H), 4.22 (d, ^2^ *J*_H–H_ = 13.56 Hz, 1 H, CH_2_Ph), 4.46 (d, ^2^ *J*_H–H_ = 12.84 Hz, 1 H, CH_2_Ph), 5.14 (d, ^2^ *J*_H–H_ = 12.78 Hz, 1 H, CH_2_Ph), 5.68 (d, ^2^ *J*_H–H_ = 13.50 Hz, 1 H, CH_2_Ph), 7.33–7.37 (m, 4 H, Ph-H), 7.40–7.44 (m, 4 H, Ph-H), 7.82 (d, ^3^ *J*_H–H_ = 6.36 Hz, 2 H,* p*-Ph-H) ppm—^13^C-NMR (150 MHz, CDCl_3_): *δ* = 14.36 (Me), 19.12 (C-4), 28.41 (C-3), 54.91 (C-5), 57.52 (CH_2_Ph), 60.22 (CH_2_Ph), 67.35 (C-2), 127.09 (Ph-C), 127.75 (Ph-C), 129.50 (Ph-CH), 129.52 (Ph-CH), 130.74 (Ph-CH), 130.76 (Ph-CH), 133.41 (Ph-CH), 133.82 (Ph-CH) ppm—MS (EI, 70 eV): *m*/*z* (%) = 175.1 [M-C_7_H_7_]^+***·***^ (5), 161.1/160.1 [M-C_8_H_10_]^+^ (5/43), 92.1/91.1 [C_7_H_7_]^+^ (6/100)—HRMS (ESI) calcd. [M]^+^ 266.19033; found 266.19018.

*tert-Butyl Benzyl(4-bromobutyl)carbamate (* ***19*** *).* NaH (3.048 g of a 55% oily dispersion, 69.8 mmol) was added to a solution of Boc protected benzylamine (4.789 g, 21.6 mmol) in DMF (dry, 50 mL) at 0°C under nitrogen atmosphere and the reaction mixture was stirred for 30 min at 0°C. 1,4-Dibromobutane (8.3 mL, 15.006 g, and 69.5 mmol) was added at 0°C and the mixture was stirred for further 3 hours. The excess of NaH was carefully hydrolysed with water and the mixture was exhaustively extracted with dichloromethane. The combined organic extracts were dried (MgSO_4_) and filtered and the solvent mixture was removed azeotropically with toluene under reduced pressure. Purification with flash column chromatography on silica gel (petroleum ether/EtOAc 10 : 1) gave product** 19** (2.019 g, 27%) and the elimination product** 21** (501.4 mg, 9%) both as colorless oils. Compound** 7**: IR (ATR-FTIR): $\tilde{\nu }$ = 2971 (w), 2931 (w), 1687 (s), 1454 (m), 1413 (m), 1363 (m), 1299 (w), 1241 (m), 1151 (s), 1108 (w), 1074 (w), 1029 (w) cm^−1^—^1^H-NMR (600 MHz, CDCl_3_, +2°C): *δ* = 1.41 (s, 9 H,* t*Bu-Me), 1.48 (s, 9 H,* t*Bu-Me), 1.56–1.66 (m, 4 H, 2-CH_2_), 1.75–1.82 (m, 4 H, 3-CH_2_), 3.12 (t, ^3^ *J*_H–H_ = 7.08 Hz, 2 H, 1-CH_2_), 3.22 (t, ^3^ *J*_H–H_ = 7.20 Hz, 2 H, 1-CH_2_), 3.34–3.39 (m, 4 H, 4-CH_2_), 4.38 (s, 2 H, CH_2_Ph), 4.43 (s, 2 H, CH_2_Ph), 7.18–7.25 (m, 6 H,* o*-Ph-H,* p*-Ph-H), 7.29–7.32 (m, 4 H,* m*-Ph-H) ppm—^13^C-NMR (150 MHz, CDCl_3_, +2°C): *δ* = 26.54 (C-2), 26.77 (C-2), 28.56 (*t*Bu-Me), 28.63 (*t*Bu-Me), 30.05 (C-3), 33.67 (C-4), 33.92 (C-4), 45.41 (C-1), 45.50 (C-1), 49.74 (CH_2_Ph), 50.39 (CH_2_Ph), 80.03 (*t*Bu-C), 80.05 (*t*Bu-C), 127.29 (Ph-CH), 127.35 (Ph-CH), 127.41 (Ph-CH), 127.85 (Ph-CH), 128.68 (*m*-Ph-CH), 128.71 (*m*-Ph-CH), 138.34 (Ph-C), 138.57 (Ph-C), 155.84 (Boc CO), 156.19 (Boc CO) ppm—MS (EI, 70 eV): *m*/*z* (%) = 287.1/286.1/285.1 [M-C_4_H_8_]^+***·***^ (30/8/29), 91.1 [C_7_H_7_]^+^ (100)—HRMS (ESI) calcd. [M+H]^+^ 342.10632; found 342.10631.

*tert-Butyl Benzyl(but-3-enyl)carbamate (* ***21*** *).* IR (ATR-FTIR): $\tilde{\nu }$ = 3068 (w), 2975 (w), 2927 (w), 1689 (s), 1643 (w), 1455 (m), 1411 (m), 1365 (m), 1294 (w), 1241 (m), 1166 (s), 1141 (s), 1027 (w), 997 (w) cm^−1^—^1^H-NMR (600 MHz, CDCl_3_, +2°C): *δ* = 1.41 (s, 9 H,* t*Bu-Me), 1.48 (s, 9 H,* t*Bu-Me), 2.18–2.21 (m, 2 H, 2-CH_2_), 2.23–2.27 (m, 2 H, 2-CH_2_), 3.13–3.16 (m, 2 H, 1-CH_2_), 3.24–3.27 (m, 2 H, 1-CH_2_), 4.39 (s, 2 H, CH_2_Ph), 4.43 (s, 2 H, CH_2_Ph), 4.97 (d, ^3^ *J*_H–H_ = 9.78 Hz, 2 H, 4-H), 5.02 (d, ^3^ *J*_H–H_ = 17.70 Hz, 2 H, 4-H), 5.66–5.77 (m, 2 H, 3-H), 7.18–7.24 (m, 6 H,* o*-Ph-H,* p*-Ph-H), 7.29–7.31 (*m*-Ph-H) ppm—^13^C-NMR (150 MHz, CDCl_3_, +2°C): *δ* = 28.56 (*t*Bu-Me), 28.62 (*t*Bu-Me), 32.59 (C-2), 33.02 (C-2), 46.13 (C-1), 46.24 (C-1), 50.04 (CH_2_Ph), 50.74 (CH_2_Ph), 79.85 (*t*Bu-C), 79.94 (*t*Bu-C), 116.74 (C-4), 116.82 (C-4), 127.26 (Ph-CH), 127.34 (Ph-CH), 127.84 (Ph-CH), 128.63 (*m*-Ph-CH), 128.68 (*m*-Ph-CH), 135.65 (C-3), 138.47 (Ph-C), 138.74 (Ph-C), 155.76 (Boc CO), 156.21 (Boc CO) ppm—MS (EI, 70 eV): *m*/*z* (%) = 207.2/206.2 [M-C_4_H_7_]^+^ (3/25), 91.1 [C_7_H_7_]^+^ (100)—HRMS (ESI) calcd. [M+Na]^+^ 284.16210; found 284.16208.

*tert-Butyl 4-Azidobutyl(benzyl)carbamate (* ***23*** *).* A reaction mixture consisting of bromine** 19** (214.3 mg, 0.63 mmol) and NaN_3_ (122.6 mg, 1.89 mmol) in DMF (dry, 2 mL) was stirred for 24 hours at 25°C. The complete conversion was determined by ^1^H-NMR spectroscopy. DMF was removed azeotropically with toluene under reduced pressure, and the residue was suspended in dichloromethane, filtered (Celite), and concentrated. Purification with flash column chromatography on silica gel (petroleum ether/EtOAc 20 : 1) gave azide** 23** (165.0 mg, 86%) as colorless oil. IR (ATR-FTIR): $\tilde{\nu }$ = 2973 (w), 2931 (w), 2869 (w), 2092 (s), 1689 (s), 1454 (m), 1413 (s), 1363 (m), 1243 (s), 1160 (s), 1126 (s), 1074 (w), 1027 (w), 1000 (w) cm^−1^—^1^H-NMR (600 MHz, CDCl_3_, +2°C): *δ* = 1.41 (s, 9 H,* t*Bu-Me), 1.48 (s, 9 H,* t*Bu-Me), 1.51–1.54 (m, 8 H, 2-CH_2_, 3-CH_2_), 3.10–3.14 (m, 2 H, 1-CH_2_), 3.20–3.25 (m, 6 H, 1-CH_2_, 4-CH_2_), 4.38 (s, 2 H, CH_2_Ph), 4.43 (s, 2 H, CH_2_Ph), 7.18–7.26 (m, 6 H,* o*-Ph-CH,* p*-Ph-CH), 7.29–7.32 (m, 4 H,* m*-Ph-CH) ppm—^13^C-NMR (150 MHz, CDCl_3_, +2°C): *δ* = 25.16 (CH_2_), 25.44 (CH_2_), 26.26 (CH_2_), 26.32 (CH_2_), 28.56 (*t*Bu-Me), 28.62 (*t*Bu-Me), 45.84 (1-CH_2_), 45.94 (1-CH_2_), 49.81 (CH_2_Ph), 50.47 (CH_2_Ph), 51.17 (C-4), 51.29 (C-4), 80.00 (*t*Bu-C), 80.06 (*t*Bu-C), 127.26 (Ph-CH), 127.35 (Ph-CH), 127.41 (Ph-CH), 127.83 (Ph-CH), 128.68 (Ph-CH), 128.71 (Ph-CH), 138.36 (Ph-C), 138.59 (Ph-C), 155.85 (Boc CO), 156.19 (Boc CO) ppm—MS (EI, 70 eV): *m*/*z* (%) = 203.1 [M-C_5_H_9_O_2_]^+^ (7), 91.0 [C_7_H_7_]^+^ (100)—HRMS (ESI) calcd. [M+Na]^+^ 327.17915; found 327.17915.

*tert-Butyl 4-Aminobutyl(benzyl)carbamate (* ***25*** *).* NaN_3_ (177.7 mg, 2.73 mmol) and PPh_3_ (482.4 mg, 1.84 mmol) were added to a solution of bromine derivative** 19** (311.9 mg, 0.91 mmol) in DMF (dry, 5 mL) and the reaction mixture was stirred for 24 hours at 25°C. Pulverised KOH (165.4 mg, 2.95 mmol) was added and stirred for further 24 hours at 25°C. DMF was removed using high vacuum, the residue was suspended in water, and the mixture was exhaustively extracted with dichloromethane. The combined organic extracts were dried (MgSO_4_), filtered, and concentrated. Purification with flash column chromatography on deactivated silica gel (DCM/MeOH 20 : 1) gave amine** 25** (119.9 mg, 47%) as colorless oil. IR (ATR-FTIR): $\tilde{\nu }$ = 2972 (w), 2925 (w), 2855 (w), 1685 (s), 1574 (w), 1494 (w), 1465 (m), 1454 (m), 1414 (s), 1390 (w), 1364 (m), 1305 (w), 1242 (m), 1163 (s), 1073 (w), 1028 (w) cm^−1^—^1^H-NMR (600 MHz, CDCl_3_, +2°C): *δ* = 1.33–1.54 (m, 26 H, 2-CH_2_, 3-CH_2_,* t*Bu-Me), 2.62–2.65 (m, 4 H, 4-CH_2_), 3.08–3.10 (m, 2 H, 1-CH_2_), 3.17–3.20 (m, 2 H, 1-CH_2_), 4.38 (s, 2 H, CH_2_Ph), 4.42 (s, 2 H, CH_2_Ph), 7.17–7.22 (m, 6 H,* o*-Ph-H,* p*-Ph-H), 7.28–7.31 (m, 4 H,* m*-Ph-H) ppm—^13^C-NMR (150 MHz, CDCl_3_, +2°C): *δ* = 25.31 (CH_2_), 25.60 (CH_2_), 28.57 (*t*Bu-Me), 28.64 (*t*Bu-Me), 30.95 (CH_2_), 31.01 (CH_2_), 42.00 (C-4), 42.30 (C-4), 46.31 (C-1), 46.55 (C-1), 49.82 (CH_2_Ph), 50.54 (CH_2_Ph), 79.76 (*t*Bu-C), 79.90 (*t*Bu-C), 127.23 (Ph-CH), 127.26 (Ph-CH), 127.32 (Ph-CH), 127.81 (Ph-CH), 128.62 (*m*-Ph-CH), 128.66 (*m*-Ph-CH), 138.54 (Ph-C), 138.78 (Ph-C), 155.82 (Boc CO), 156.26 (Boc CO) ppm—MS (EI, 70 eV): *m*/*z* (%) = 279.2/278.2 [M]^+***·***^ (1/3), 223.1/222.1 [M-C_4_H_8_]^+***·***^ (3/16), 178.1/177.1 [M-C_5_H_9_O_2_]^+^ (2/13), 91.1 [C_7_H_7_]^+^ (100)—HRMS (ESI) calcd. [M+H]^+^ 279.20670; found 279.20671.

*tert-Butyl Benzyl(10-bromodecyl)carbamate (* ***20*** *).* NaH (2.498 g of a 55% oily dispersion, 57.2 mmol) was added to a solution of Boc protected benzylamine (4.011 g, 19.4 mmol) in DMF (dry, 50 mL) at 0°C under nitrogen atmosphere and the reaction mixture was stirred for 30 min at 0°C. 1,10-Dibromodecane (13.0 mL, 17.355 g, and 57.8 mmol) was added at 0°C and the mixture was stirred for further 3.5 hours. The excess of NaH was hydrolysed carefully using water and the mixture was exhaustively extracted with dichloromethane. The combined organic extracts were dried (MgSO_4_) and the solvent mixture was removed azeotropically with toluene under reduced pressure. Purification with flash column chromatography on silica gel (petroleum ether/EtOAc 40 : 1) gave product** 20** (3.604 g, 44%) and the elimination product** 22** (304.6 mg, 5%) both as colorless oils. Compound** 20**: IR (ATR-FTIR): $\tilde{\nu }$ = 2971 (w), 2925 (m), 2854 (w), 1689 (s), 1455 (m), 1413 (m), 1363 (m), 1303 (w), 1240 (m), 1164 (s), 1027 (w) cm^−1^—^1^H-NMR (600 MHz, CDCl_3_, +2°C): *δ* = 1.16–1.28 (m, 20 H, 3-CH_2_, 4-CH_2_, 5-CH_2_, 6-CH_2,_ 7-CH_2_), 1.37–1.47 (m, 26 H,* t*Bu-Me, 2-CH_2_, 8-CH_2_), 1.79–1.83 (m, 4 H, 9-CH_2_), 3.06 (t, ^3^ *J*_H–H_ = 7.26 Hz, 1-CH_2_), 3.16 (t, ^3^ *J*_H–H_ = 7.56 Hz, 1-CH_2_), 3.39 (t, ^3^ *J*_H–H_ = 6.84 Hz, 4 H, 10-CH_2_), 4.37 (s, 2 H, CH_2_Ph), 4.42 (s, 2 H, CH_2_Ph), 7.18–7.24 (m, 6 H,* o*-Ph-H,* p*-Ph-H), 7.29–7.31 (m, 4 H,* m*-Ph-H) ppm—^13^C-NMR (150 MHz, CDCl_3_, +2°C): *δ* = 26.94 (C-3), 27.02 (C-3), 28.00 (C-2), 28.15 (C-2), 28.32 (C-8), 28.58 (*t*Bu-Me), 28.65 (*t*Bu-Me), 28.92 (CH_2_), 29.44 (CH_2_), 29.53 (CH_2_), 29.55 (CH_2_), 29.65 (CH_2_), 32.95 (C-9), 32.96 (C-9), 34.49 (C-10), 34.52 (C-10), 46.42 (C-1), 46.75 (C-1), 49.73 (CH_2_Ph), 50.43 (CH_2_Ph), 79.63 (*t*Bu-C), 79.77 (*t*Bu-C), 127.19 (Ph-CH), 127.21 (Ph-CH), 127.25 (Ph-CH), 127.79 (Ph-CH), 128.59 (*m*-Ph-CH), 128.63 (*m*-Ph-CH), 138.63 (Ph-C), 138.92 (Ph-C), 155.81 (Boc CO), 156.35 (Boc CO) ppm—MS (EI, 70 eV): *m*/*z* (%) = 371.2/370.2/369.2 [M-C_4_H_8_]^+***·***^ (21/8/20), 91.1 [C_7_H_7_]^+^ (100)—HRMS (ESI) calcd. [M+Na]^+^ 448.18216; found 448.18218.

*tert-Butyl Benzyl(dec-9-enyl)carbamate (* ***22*** *).* IR (ATR-FTIR): $\tilde{\nu }$ = 2973 (w), 2925 (m), 2854 (w), 1691 (s), 1641 (w), 1455 (m), 1413 (m), 1363 (m), 1303 (w), 1241 (m), 1164 (s), 1029 (w), 993 (w) cm^−1^—^1^H-NMR (600 MHz, CDCl_3_, +2°C): *δ* = 1.16–1.25 (m, 16 H, 3-CH_2_, 4-CH_2_, 5-CH_2_, 6-CH_2_), 1.30–1.35 (m, 4 H, 7-CH_2_), 1.40–1.47 (m, 22 H,* t*Bu-Me, 2-CH_2_), 1.97–2.03 (m, 4 H, 8-CH_2_), 3.06 (t, ^3^ *J*_H–H_ = 7.26 Hz, 1-CH_2_), 3.16 (t, ^3^ *J*_H–H_ = 7.56 Hz, 1-CH_2_), 4.37 (s, 2 H, CH_2_Ph), 4.42 (s, 2 H, CH_2_Ph), 4.90 (d, ^3^ *J*_H–H_ = 9.96 Hz, 2 H, 10-H), 4.97 (d, ^3^ *J*_H–H_ = 17.16 Hz, 2 H, 10-H), 5.75–5.82 (m, 2 H, 9-H), 7.18–7.24 (m, 6 H,* o*-Ph-H,* p*-Ph-H), 7.28–7.31 (m, 4 H,* m*-Ph-H) ppm—^13^C-NMR (150 MHz, CDCl_3_, +2°C): *δ* = 26.93 (CH_2_), 27.03 (CH_2_), 28.00 (C-2), 28.14 (C-2), 28.58 (*t*Bu-Me), 28.64 (*t*Bu-Me), 29.04 (C-7), 29.23 (CH_2_), 29.43 (CH_2_), 29.53 (CH_2_), 29.61 (CH_2_), 34.01 (C-8), 46.42 (C-1), 46.76 (C-1), 49.72 (CH_2_Ph), 50.43 (CH_2_Ph), 79.62 (*t*Bu-C), 79.76 (*t*Bu-C), 114.32 (C-10), 114.37 (C-10), 127.18 (Ph-CH), 127.22 (Ph-CH), 127.25 (Ph-CH), 127.79 (Ph-CH), 128.58 (*m*-Ph-CH), 128.63 (*m*-Ph-CH), 138.63 (Ph-C), 138.92 (Ph-C), 139.41 (C-9), 139.47 (C-9), 155.80 (Boc CO), 156.36 (Boc CO) ppm—MS (EI, 70 eV): *m*/*z* (%) = 292.4/291.4/290.4 [M-C_4_H_8_+H]^+^ (1/8/40), 247.3/246.3/245.3 [M-Boc]^+***·***^ (4/21/1), 91.1 [Bn]^+^ (100)—HRMS (ESI) calcd. [M+Na]^+^ 368.25600; found 368.25601 [M+Na]^+^.

*tert-Butyl 5-Azidopentyl(benzyl)carbamate (* ***24*** *).* A suspension consisting of bromine derivative** 20** (249.0 mg, 0.58 mmol) and NaN_3_ (115.2 mg, 1.77 mmol) in DMF (dry, 2 mL) was stirred for 24 hours at 25°C. The complete conversion was determined by ^1^H-NMR spectroscopy. DMF was removed azeotropically with toluene under reduced pressure, and the residue was suspended in dichloromethane, filtered (Celite), and concentrated. Purification with flash column chromatography on silica gel (petroleum ether/EtOAc 20 : 1) gave azide** 24** (214.9 mg, 95%) as colorless oil. IR (ATR-FTIR): $\tilde{\nu }$ = 2973 (w), 2925 (w), 2854 (w), 2092 (m), 1691 (s), 1455 (m), 1413 (m), 1363 (m), 1241 (m), 1164 (s), 1093 (w), 1029 (w) cm^−1^—^1^H-NMR (600 MHz, CDCl_3_, +2°C): *δ* = 1.16–1.26 (m, 24 H, 2-CH_2_, 3-CH_2_, 4-CH_2_, 5-CH_2_, 6-CH_2_, 7-CH_2_), 1.29–1.35 (m, 4 H, 8-CH_2_), 1.40 (s, 9 H,* t*Bu-Me), 1.47 (s, 9 H,* t*Bu-Me), 1.55–1.57 (m, 4 H, 9-CH_2_), 3.06 (t, ^3^ *J*_H–H_ = 7.20 Hz, 2 H, 1-CH_2_), 3.16 (t, ^3^ *J*_H–H_ = 7.56 Hz, 2 H, 1-CH_2_), 3.23 (t, ^3^ *J*_H–H_ = 6.84 Hz, 4 H, 10-CH_2_), 4.37 (s, 2 H, CH_2_Ph), 4.42 (s, 2 H, CH_2_Ph), 7.18–7.24 (m, 6 H,* o*-Ph-H,* p*-Ph-H), 7.29–7.31 (m, 4 H,* m*-Ph-H) ppm—^13^C-NMR (150 MHz, CDCl_3_, +2°C): *δ* = 26.87 (C-8), 26.95 (CH_2_), 27.03 (CH_2_), 28.00 (C-2), 28.15 (C-2), 28.59 (*t*Bu-Me), 28.65 (*t*Bu-Me), 28.99 (C-9), 29.31 (CH_2_), 29.33 (CH_2_), 29.46 (CH_2_), 29.53 (CH_2_), 29.58 (CH_2_), 29.61 (CH_2_), 29.67 (CH_2_), 46.41 (C-1), 46.75 (C-1), 49.72 (CH_2_Ph), 50.43 (CH_2_Ph), 51.61 (C-10), 79.62 (*t*Bu-C), 79.77 (*t*Bu-C), 127.19 (Ph-CH), 127.22 (Ph-CH), 127.26 (Ph-CH), 127.79 (Ph-CH), 128.59 (*m*-Ph-CH), 128.63 (*m*-Ph-CH), 138.64 (Ph-C), 138.93 (Ph-C), 155.81 (Boc CO), 156.35 (Boc CO) ppm—MS (EI, 70 eV): *m*/*z* (%) = 288.2/287.1 [M-C_5_H_9_O_2_]^+^ (2/11), 91.0 [C_7_H_7_]^+^ (100)—HRMS (ESI) calcd. [M+H]^+^ 389.29110; found 389.29110.

*tert-Butyl 10-Aminodecyl(benzyl)carbamate (* ***26*** *).* NaN_3_ (149.2 mg, 2.30 mmol) and PPh_3_ (402.6 mg, 1.53 mmol) were added to a solution of bromine derivative** 20** (321.5 mg, 0.75 mmol) in DMF (dry, 5 mL) and the reaction mixture was stirred for 24 hours at 25°C. Pulverized KOH (127.1 mg, 2.27 mmol) was added and the mixture was stirred for further 24 hours at 25°C. DMF was removed using high vacuum, the residue was suspended in water, and the mixture was exhaustively extracted with dichloromethane. The combined organic extracts were dried (MgSO_4_), filtered, and concentrated. Purification on deactivated silica gel (DCM/MeOH 20 : 1) gave compound** 26 **(105.9 mg, 39%) as colorless oil. IR (ATR-FTIR): $\tilde{\nu }$ = 2970 (w), 2923 (m), 2852 (m), 1690 (s), 1570 (w), 1494 (w), 1454 (m), 1414 (m), 1390 (w), 1364 (m), 1305 (w), 1240 (m), 1164 (s), 1028 (w) cm^−1^—^1^H-NMR (600 MHz, CDCl_3_, +2°C): *δ* = 1.16–1.27 (m, 24 H, 2-CH_2_, 3-CH_2_, 4-CH_2_, 5-CH_2_, 6-CH_2_, 7-CH_2_), 1.39–1.46 (m, 26 H,* t*Bu-Me, 8-CH_2_, 9-CH_2_), 1.72 (s, br, 4 H, NH_2_), 2.63–2.65 (m, 4 H, 10-CH_2_), 3.05–3.07 (m, 2 H, 1-CH_2_), 3.14–3.17 (m, 2 H, 1-CH_2_), 4.37 (s, 2 H, CH_2_Ph), 4.42 (s, 2 H, CH_2_Ph), 7.17–7.23 (m, 6 H,* o*-Ph-H,* p*-Ph-H), 7.28–7.30 (m, 4 H,* m*-Ph-H) ppm—^13^C-NMR (150 MHz, CDCl_3_, +2°C): *δ* = 26.95 (CH_2_), 27.04 (CH_2_), 28.00 (CH_2_), 28.14 (CH_2_), 28.57 (*t*Bu-Me), 28.64 (*t*Bu-Me), 29.48 (CH_2_), 29.56 (CH_2_), 29.64 (CH_2_), 29.66 (CH_2_), 29.73 (CH_2_), 33.84 (9-CH_2_), 42.32 (10-CH_2_), 46.41 (1-CH_2_), 46.76 (1-CH_2_), 49.71 (CH_2_Ph), 50.42 (CH_2_Ph), 79.60 (*t*Bu-C), 79.75 (*t*Bu-C), 127.17 (Ph-CH), 127.21 (Ph-CH), 127.24 (Ph-CH), 127.78 (Ph-CH), 128.58 (*m*-Ph-CH), 128.62 (*m*-Ph-CH), 138.63 (Ph-C), 138.92 (Ph-C), 155.79 (Boc CO), 156.35 (Boc CO) ppm—MS (EI, 70 eV): *m*/*z* (%) = 363.3/362.3 [M]^+***·***^ (2/5), 306.2 [M-C_4_H_8_]^+***·***^ (2), 263.2/262.2/261.2 [M-C_5_H_9_O_2_]^+^ (2/13/46), 91.1 [C_7_H_7_]^+^ (100)—HRMS (ESI) calcd. [M+H]^+^ 363.30060; found 363.30060.

*N-Benzyl-3,3-dimethylbutanamide (* ***31*** *).* 3,3-Dimethylbutanoyl chloride (7.9 ml, 7.655 g, and 56.9 mmol) was added to a solution of benzylamine (5.073 g, 47.3 mmol) and triethylamine (9.9 mL, 7.187 g, and 71.0 mmol) in DCM (dry, 60 mL) at 0°C. The reaction mixture was stirred for 15 min at 0°C, aqueous NaOH solution was added, and the mixture was exhaustively extracted with dichloromethane. The combined organic extracts were dried (MgSO_4_), filtered, and concentrated. The residue was purified using flash column chromatography on silica gel (petroleum ether/EtOAc 5 : 1) to give product** 31** (9.162, 94%) as colorless solid. Mp 72°C (petroleum ether/EtOAc)—IR (ATR-FTIR): $\tilde{\nu }$ = 3278 (w, br), 3060 (w), 3028 (w), 2954 (w), 2865 (w), 2357 (w), 2335 (w), 1633 (s), 1542 (s), 1497 (m), 1461 (m), 1391 (w), 1364 (m), 1338 (m), 1266 (m), 1234 (m), 1202 (w), 1148 (w), 1072 (w), 1030 (w), 1009 (w) cm^−1^—^1^H-NMR (600 MHz, CDCl_3_): *δ* = 1.03 (s, 9 H,* t*Bu-Me), 2.07 (s, 2 H, 2-CH_2_), 4.41 (d, ^3^ *J*_H–H_ = 5.64 Hz, 2 H, CH_2_Ph), 5.68 (s, br, 1 H, NH), 7.25–7.27 (m, 3 H,* o*-Ph-H,* p*-Ph-H), 7.30–7.32 (m, 2 H,* m*-Ph-H) ppm—^13^C-NMR (150 MHz, CDCl_3_): *δ* = 30.07 (*t*Bu-Me), 31.17 (*t*Bu-C), 43.82 (CH_2_Ph), 50.82 (C-2), 127.69 (*p*-Ph-C), 128.12 (*o*-Ph-C), 128.90 (*m*-Ph-C), 138.66 (Ph-C), 171.74 (amide CO) ppm—MS (EI, 70 eV): *m*/*z* (%) = 206.4/205.4 [M]^+***·***^ (8/48), 149.2/148.2 [M-C_4_H_9_]^+^ (80/48), 92.1/91.1 [C_7_H_7_]^+^ (14/100)—HRMS (ESI) calc. [M+H]^+^ 206.15394; found 206.15394.

*N-Benzyl-N-(4*′*-bromopentyl)-3,3-dimethylbutanamide (* ***32*** *).* NaH (943.2 mg of a 55% oily dispersion, 21.6 mmol) was added to a solution of** 31** (1.486 g, 7.24 mmol) in DMF (dry, 20 mL) at 0°C under nitrogen atmosphere and the reaction mixture was stirred for 30 min at 0°C. 1,4-Dibromopentane (2.2 mL, 3.711 g, and 16.1 mmol) was added at 0°C, and the reaction mixture was allowed to warm up to 25°C and was stirred for further 6 hours. After addition of aqueous NaCl solution the mixture was exhaustively extracted with dichloromethane, the combined organic extracts were dried (MgSO_4_) and filtered, and the solvent mixture was removed azeotropically with toluene under reduced pressure. Purification with flash column chromatography on silica gel (petroleum ether/EtOAc 10 : 1) gave compound** 32** (564.4 mg, 22%) and compound** 33** (93.8 mg, 5%) both as colorless oil. Compound** 32**: IR (ATR-FTIR): $\tilde{\nu }$ = 2951 (m), 2865 (w), 1635 (s), 1495 (w), 1451 (m), 1416 (m), 1361 (m), 1324 (w), 1229 (m), 1186 (m), 1152 (m), 1121 (m), 1078 (w), 1028 (m), 957 (m) cm^−1^—^1^H-NMR (600 MHz, CDCl_3_): *δ* = 1.04 (s, 9 H,* t*Bu-Me,** A**), 1.07 (s, 9 H,* t*Bu-Me,** B**), 1.66 (d, ^3^ *J*_H–H_ = 6.66 Hz, 6 H, Me,** A**,** B**), 1.69–1.77 (m, 8 H, 2′-CH_2_, 3′-CH_2_,** A**,** B**), 2.24 (dd, ^2^ *J*_H–H_ = 14.40 Hz, 2 H, 2-CH_2_,** A**), 2.29 (dd, ^2^ *J*_H–H_ = 14.22 Hz, 2 H, 2-CH_2_,** B**), 3.16–3.27 (m, 2 H, 1′-CH_2_,** B**), 3.28–3.33 (m, 1 H, 1′-CH_2_,** A**), 3.37–3.41 (m, 1 H, 1′-CH_2_,** A**), 4.02–4.05 (m, 1 H, 4′-H,** B**), 4.10–4.13 (m, 1 H, 4′-H,** A**), 4.55 (dd, ^2^ *J*_H–H_ = 17.04 Hz, 2 H, CH_2_Ph,** A**), 4.60 (s, 2 H, CH_2_Ph,** B**), 7.14 (d, ^3^ *J*_H–H_ = 7.50 Hz, 2 H,* o*-Ph-H,** A**), 7.23–7.25 (m, 3 H,* o*-Ph-H,* p*-Ph-H,** B**), 7.26–7.30 (m, 3 H,* m*-Ph-H,* p*-Ph-H,** A**), 7.33–7.35 (m, 2 H,* m*-Ph-H) ppm—^13^C-NMR (150 MHz, CDCl_3_): *δ* = 26.09 (CH_2_,** A**), 26.73 (Me,** A**,** B**), 26.94 (CH_2_,** B**), 30.26 (*t*Bu-Me,** A**), 30.35 (*t*Bu-Me,** B**), 31.73 (*t*Bu-C,** A**), 31.84 (*t*Bu-C,** B**), 38.15 (CH_2_,** B**), 38.54 (CH_2_,** A**), 44.82 (C-2,** B**), 45.06 (C-2,** A**), 47.00 (C-1′,** B**), 48.20 (CH_2_Ph,** B**), 50.74 (C-4′,** B**), 51.57 (C-4′,** A**), 51.84 (CH_2_Ph,** A**), 126.54 (*o*-Ph-CH,** A**), 127.46 (*p*-Ph-CH,** B**), 127.80 (*p*-Ph-CH,** A**), 128.32 (*o*-Ph-CH,** B**), 128.75 (*m*-Ph-CH), 129.10 (*m*-Ph-CH), 137.24 (Ph-C,** A**), 138.31 (Ph-C,** B**), 172.13 (C-1,** B**), 172.58 (C-1,** A**) ppm—MS (EI, 70 eV): *m*/*z* (%) = 355.1/354.1/353.1 [M]^+***·***^ (10/2/10), 275.2/274.2 [M-Br]^+^ (12/55), 219.2/218.2 [M-C_4_H_8_Br]^+^ (8/50), 121.1/120.1 [C_8_H_10_N]^+^ (6/61), 92.0/91.0 [C_7_H_7_]^+^ (9/100)—HRMS (ESI) calcd. [M+H]^+^ 354.14270; found 354.14272.

*(E)-N-Benzyl-3,3-dimethyl-N-(pent-3*′*-enyl)butanamide (* ***33*** *).* IR (ATR-FTIR): $\tilde{\nu }$ = 2954 (m), 2868 (w), 1735 (w), 1642 (s), 1495 (w), 1452 (m), 1414 (m), 1388 (w), 1360 (m), 1226 (m), 1183 (m), 1129 (w), 1078 (w), 1027 (w), 969 (m) cm^−1^—^1^H-NMR (600 MHz, CDCl_3_): *δ* = 0.93–0.98 (m, 6 H, Me,** A**,** B**), 1.04 (s, 9 H,* t*Bu-Me,** B**), 1.07 (s, 9 H,* t*Bu-Me,** A**), 1.99–2.05 (m, 4 H, 2′-CH_2_,** A**,** B**), 2.26 (s, 2 H, 2-CH_2_,** B**), 2.29 (s, 2 H, 2-CH_2_,** A**), 3.77 (d, ^3^ *J*_H–H_ = 4.44 Hz, 2 H, 1′-CH_2_,** A**), 3.92 (d, ^3^ *J*_H–H_ = 5.58 Hz, 1′-CH_2_,** B**), 4.50 (s, 2 H, CH_2_Ph,** B**), 4.56 (s, 2 H, CH_2_Ph,** A**), 5.28–5.30 (m, 1 H, 3′-H,** A**), 5.36–5.38 (m, 1 H, 3′-H,** B**), 5.52–5.58 (m, 2 H, 4′-H,** A**,** B**), 7.13-7.14 (m, 2 H, Ph-H), 7.22–7.33 (m, 8 H, Ph-H) ppm—^13^C-NMR (150 MHz, CDCl_3_): *δ* = 13.68 (Me,** A**,** B**), 25.45 (C-2′), 30.25 (*t*Bu-Me,** B**), 30.33 (*t*Bu-Me,** A**), 31.71 (*t*Bu-C,** A**,** B**), 44.81 (C-2,** A**), 45.00 (C-2,** B**), 47.23 (C-1′,** B**), 47.85 (CH_2_Ph,** A**), 49.38 (C-1′,** A**), 50.63 (CH_2_Ph,** B**), 123.49 (C-3′,** A**), 124.04 (C-3′,** B**), 126.62 (Ph-CH), 127.41 (Ph-CH), 127.64 (Ph-CH), 128.46 (Ph-CH), 128.67 (Ph-CH), 129.00 (Ph-CH), 135.63 (C-4′,** A**), 136.21 (C-4′,** B**), 137.38 (Ph-C,** B**), 138.36 (Ph-C,** A**), 172.32 (amide CO,** B**), 172.38 (amide CO,** A**) ppm—MS (EI, 70 eV): *m*/*z* (%) = 274.2/273.2 [M]^+***·***^ (7/24), 259.2/258.2 [C_17_H_24_NO]^+^ (3/16), 205.1/204.1 [C_13_H_18_NO]^+^ (8/56), 183.1/182.1 [M-C_7_H_7_]^+^ (5/43), 107.0/106.0 [C_7_H_8_N]^+^ (17/97), 92.0/91.0 [C_7_H_7_]^+^ (10/100), 85.0/84.0 [C_5_H_10_N]^+^ (6/85). HRMS (ESI) calcd. [M+H]^+^ 274.21654; found 274.21655.

*N-(4*′*-Azidopentyl)-N-benzyl-3,3-dimethylbutanamide (* ***34*** *).* A suspension of the bromine compound** 32** (199.2 mg, 0.56 mmol) and NaN_3_ (108.4 mg, 1.67 mmol) in DMF (dry, 5 mL) was stirred at 25°C for 24 hours. DMF was removed azeotropically with toluene under reduced pressure, and the residue was suspended in dichloromethane, filtered (Celite), and concentrated. Purification with flash column chromatography on silica gel (petroleum ether/EtOAc 10 : 1) gave compound** 34** (169.1 mg, 95%) as colorless oil. IR (ATR-FTIR): $\tilde{\nu }$ = 2950 (m), 2866 (w), 2097 (s), 1636 (s), 1495 (w), 1452 (m), 1416 (m), 1378 (m), 1361 (m), 1327 (m), 1233 (m), 1186 (m), 1120 (m), 1078 (w), 1028 (w), 954 (w) cm^−1^—^1^H-NMR (600 MHz, CDCl_3_): *δ* = 1.04 (s, 9 H,* t*Bu,** A**), 1.07 (s, 9 H,* t*Bu,** B**), 1.22 (t, ^3^ *J*_H–H_ = 6.68 Hz, 6 H, Me), 1.36–1.39 (m, 2 H, 3′-CH_2_,** B**), 1.41–1.44 (m, 2 H, 3′-CH_2_,** A**), 1.52–1.67 (m, 4 H, 2′-CH_2_), 2.24 (s, 2 H, 2-CH_2_,** A**), 2.28 (s, 2 H, 2-CH_2_,** B**), 3.21 (t, ^3^ *J*_H–H_ = 7.68 Hz, 2 H, 1-CH_2_,** B**), 3.29–3.44 (m, 4 H, 1-CH_2_,** A**; 4′-H,** A**,** B**), 4.54 (s, 2 H, CH_2_Ph,** A**), 4.59 (s, 2 H, CH_2_Ph,** B**), 7.13-7.14 (m, 2 H, Ph-H), 7.22–7.24 (m, 3 H, Ph-H), 7.26–7.30 (m, 3 H, Ph-H), 7.33–7.35 (m, 2 H, Ph-H) ppm—^13^C-NMR (150 MHz, CDCl_3_): *δ* = 19.68 (Me), 24.43 (C-2′,** A**), 25.32 (C-2′,** B**), 30.24 (*t*Bu-Me,** A**), 30.35 (*t*Bu-Me,** B**), 31.73 (*t*Bu-C,** A**), 31.83 (*t*Bu-C,** B**), 33.51 (C-3′,** B**), 33.77 (C-3′,** A**), 44.83 (C-2,** B**), 45.05 (C-2,** A**), 45.61 (C-1′,** A**), 47.44 (C-1′,** B**), 48.25 (CH_2_Ph,** B**), 51.95 (CH_2_Ph,** A**), 57.69 (C-4′,** B**), 57.89 (C-4′,** A**), 126.52 (Ph-CH), 127.46 (Ph-CH), 127.81 (Ph-CH), 128.29 (Ph-CH), 128.76 (Ph-CH), 129.10 (Ph-CH), 137.24 (Ph-C,** A**), 138.31 (Ph-C,** B**), 172.12 (amide CO,** B**), 172.58 (amide CO,** A**) ppm—MS (EI, 70 eV): *m*/*z* (%) = 219.2/218.2/217.2 [M-C_6_H_11_O]^+^ (3/17/59), 121.1/120.1 [C_8_H_10_N]^+^ (5/52), 107.1/106.1 [C_7_H_8_N]^+^ (4/17), 92.1/91.0 [C_7_H_7_]^+^ (9/100)—HRMS (ESI) calcd. [M+H]^+^ 317.23359; found 317.23360.

*N-(4-Aminopentyl)-N-benzyl-3,3-dimethylbutanamide (* ***35*** *).* PPh_3_ (175.2 mg, 0.67 mmol) was added to a solution of azide** 34 **(175.0 mg, 0.55 mmol) in MeOH (dry, 10 mL) and stirred over night at 25°C. The suspension was concentrated and purified with flash column chromatography on deactivated silica gel (DCM/MeOH 20 : 1) to obtain compound** 35** (143.2 mg, 0.49 mmol, and 89%) as colorless oil. IR (ATR-FTIR): $\tilde{\nu }$ = 2950 (m), 2865 (m), 2360 (w), 2341 (w), 1633 (s), 1584 (m), 1494 (w), 1463 (m), 1451 (m), 1388 (m), 1361 (m), 1325 (w), 1277 (w), 1230 (m), 1186 (m), 1153 (w), 1120 (m), 1078 (w), 1028 (w), 955 (w), 892 (w), 815 (w), 730 (m), 699 (m), 633 (w), 619 (w) cm^−1^—^1^H-NMR (600 MHz, CDCl_3_): *δ* = 1.03–1.06 (m, 24 H,* t*Bu-Me, Me), 1.23–1.27 (m, 2 H, 3′-CH_2_,** A**), 1.29–1.34 (m, 2 H, 3′-CH_2_,** B**), 1.47–1.61 (m, 4 H, 2′-CH_2_), 2.23 (s, 2 H, 2-CH_2_,** B**), 2.28 (s, 2 H, 2-CH_2_,** A**), 2.83–2.87 (m, 1 H, 4′-H,** A**), 2.88–2.94 (m, 1 H, 4′-H,** B**), 3.20 (t, ^3^ *J*_H–H_ = 7.74 Hz, 2 H, 1′-CH_2_,** A**), 3.25–3.30 (m, 1 H, 1′-CH_2_,** B**), 3.32–3.37 (m, 1 H, 1′-CH_2_,** B**), 4.54 (s, 2 H, CH_2_Ph,** B**), 4.59 (s, 2 H, CH_2_Ph,** A**), 7.16 (d, ^3^ *J*_H–H_ = 7.38 Hz, 2 H, Ph-H), 7.22–7.24 (m, 3 H, Ph-H), 7.25–7.29 (m, 3 H, Ph-H), 7.32–7.34 (m, 2 H, Ph-H) ppm—^13^C-NMR (150 MHz, CDCl_3_): *δ* = 23.65 (Me,** B**), 24.02 (Me,** A**), 24.62 (C-2′,** B**), 25.69 (C-2′,** A**), 30.25 (*t*Bu-Me,** B**), 30.35 (*t*Bu-Me,** A**), 31.71 (*t*Bu-C,** B**), 31.80 (*t*Bu-C,** A**), 36.87 (C-3′,** B**), 36.94 (C-3′,** A**), 44.83 (C-2,** A**), 45.06 (C-2,** B**), 46.25 (C-1′,** B**), 46.96 (C-4′,** A**), 47.04 (C-4′,** B**), 47.91 (C-1′,** A**), 48.26 (CH_2_Ph,** A**), 52.11 (CH_2_Ph,** B**), 126.51 (Ph-CH), 127.38 (Ph-CH), 127.75 (Ph-CH), 128.26 (Ph-CH), 128.70 (Ph-CH), 129.06 (Ph-CH), 137.34 (Ph-C,** B**), 138.43 (Ph-C,** A**), 172.15 (amide CO,** A**), 172.54 (amide CO,** B**) ppm—MS (EI, 70 eV): *m*/*z* (%) = 290.2 [M]^+***·***^ (4), 275.2 [M-CH_3_]^+^ (8), 248.2/247.2 [M-C_3_H_7_]^+^ (9/46), 192.2/191.2 [M-C_6_H_11_O]^+^ (10/68), 121.1/120.1 [C_8_H_10_N]^+^ (3/36), 92.1/91.1 [C_7_H_7_]^+^ (10/100)—HRMS (ESI) calcd. [M+H]^+^ 291.24309; found 291.24319.

*2-(4*′*-Bromopentyl)isoindoline-1,3-dione (* ***36*** *) [33, 38].* (*rac*)-1,4-Dibromopentane (10.122 g, 0.044 mol, and 6.0 mL) was added to a suspension of potassium phthalimide (6.050 g, 0.033 mol) in acetone (35 mL) at 25°C and heated up to 80°C for 24 hours. The suspension was filtered and concentrated and the residue was distilled to give compound** 36** (6.806 g, 0.023 mol, and 70%) as light yellow oil. Bp 180°C (3.4*·*10^−1^ mbar)—IR (ATR-FTIR): $\tilde{\nu }$ = 2955 (w), 2938 (w), 2864 (w), 1771 (w), 1702 (s), 1615 (w), 1465 (w), 1433 (m), 1394 (s), 1376 (m), 1360 (s), 1322 (m), 1303 (w), 1286 (w), 1264 (m), 1243 (m), 1186 (m), 1169 (w), 1157 (w), 1138 (w), 1127 (m), 1083 (m), 1034 (s), 994 (w), 972 (w), 926 (m), 897 (w), 882 (m), 831 (w), 795 (w), 776 (m), 712 (s), 693 (m), 648 (s), 604 (m) cm^−1^—^1^H-NMR (400 MHz, CDCl_3_): *δ* = 1.68 (d, ^3^ *J*_H–H_ = 6.68 Hz, Me), 1.76–1.95 (m, 4 H, 2′-CH_2_, 3′-CH_2_), 3.68–3.72 (m, 2 H, 1′-CH_2_), 4.10–4.18 (m, 1 H, 4′-CH), 7.69–7.71 (m, 2 H, ar-H), 7.82–7.84 (m, 2 H, ar-H) ppm—^13^C-NMR (100 MHz, CDCl_3_): *δ* = 26.66, 27.22, 37.39, 38.31, 50.69 (C-4′), 123.48 (ar-CH), 132.30 (ar-Cq), 134.19 (ar-CH), 168.59 (imide CO) ppm—^15^N-NMR: (40.5 MHz, DMSO-d_6_): −219.0 (N-2) ppm—MS (EI, 70 eV): *m*/*z* (%) = 295.1 [M]^+***·***^ (4), 216.1 [M-Br]^+^ (74), 160.0 [M-(CH_2_)_2_CHBrCH_3_]^+^ (100)—CHN calcd. for C_13_H_14_BrNO_2_: C: 52.72 H: 4.76 N: 4.73; found C: 53.64 H: 4.66 N: 4.96.

*2,*2′*-(Pentane-1*′′*,4*′′*-diyl)diisoindoline-1,3-dione (* ***31*** *).* The distillation residue was further purified using flash column chromatography on silica gel (using gradients from petroleum ether/EtOAc 5 : 1 to EE 100% to CH_2_Cl_2_/MeOH 10 : 1) and the obtained solid was recrystallized (DCM/petroleum ether) to obtain compound** 31** (599.2 mg, 1.65 mmol, 5%) as light yellow crystals. Mp 147°C (DCM/petroleum ether)—IR (ATR-FTIR): $\tilde{\nu }$ = 2971 (w), 2933 (w), 2877 (w), 1769 (w), 1757 (w), 1696 (s), 1611 (w), 1463 (m), 1437 (w), 1391 (m), 1378 (m), 1357 (m), 1327 (m), 1284 (w), 1265 (w), 1243 (w), 1186 (w), 1168 (w), 1139 (w), 1113 (w), 1085 (w), 1059 (s), 1015 (m), 982 (m), 970 (m), 935 (w), 886 (m), 846 (w), 827 (w), 797 (m), 755 (w), 718 (s), 709 (s), 614 (m) cm^−1^—^1^H-NMR (600 MHz, CDCl_3_): *δ* = 1.45 (d, ^3^ *J*_H–H_ = 6.96 Hz, 3 H, Me), 1.53–1.69 (m, 2 H, 2′′-CH_2_), 1.73–1.79 (m, 1 H, 3′′-H), 2.07–2.14 (m, 1 H, 3′′-H), 3.62–3.71 (m, 2 H, 1′′-CH_2_), 4.34–4.40 (m, 1 H, 4′′-H), 7.66–7.68 (m, 4 H, 5-H, 6-H, 5′-H, 6′-H), 7.77–7.79 (m, 4 H, 4-H, 7-H, 4′-H, 7′-H) ppm—^13^C-NMR (150 MHz, CDCl_3_): *δ* = 18.86 (Me), 26.17 (C-2′′), 31.10 (C-3′′), 37.65 (C-1′′), 47.13 (C-4′′), 123.32 (CH), 123.41 (ar-CH), 132.10 (ar-Cq), 132.27 (ar-Cq), 134.07 (ar-CH), 134.10 (ar-CH), 168.56 (imide CO), 168.66 (imide CO) ppm—MS (EI, 70 eV): *m*/*z* (%) = 363.3/362.3 [M]^+***·***^ (6/26), 175.1/174.1 [C_10_H_8_NO_2_]^+^ (14/100), 161.1/160.1 [C_9_H_6_NO_2_]^+^ (5/30)—HRMS (ESI) calc. [M+H]^+^ 363.13393; found 363.13394.

*(E)-2-(Pent-3*′*-enyl)isoindoline-1,3-dione (* ***37*** *).* The elimination product** 37** was also obtained within the synthesis of** 36** as colorless crystals (with varying yields). Mp. 64–67°C (petroleum ether/EtOAc)—IR (ATR-FTIR): $\tilde{\nu }$ = 3063 (w), 3022 (w), 2965 (w), 2938 (w), 2914 (w), 2853 (w), 2159 (w, br), 2026 (w), 1976 (w), 1766 (m), 1698 (s), 1614 (m), 1595 (w), 1467 (w), 1445 (m), 1433 (m), 1394 (s), 1359 (m), 1331 (m), 1289 (w), 1265 (w), 1239 (w), 1188 (m), 1172 (m), 1150 (w), 1061 (m), 1028 (w), 990 (m), 961 (m), 903 (w), 865 (m), 790 (m), 750 (w), 717 (s), 621 (m) cm^−1^—^1^H-NMR (400 MHz, CDCl_3_): *δ* = 1.56–1.58 (m, 3 H, Me), 2.31–2.36 (m, 2 H, 2′-CH_2_), 3.67–3.71 (m, 2 H, 1′-CH_2_), 5.34–5.51 (m, 2 H, 3′-H, 4′-H), 7.67–7.69 (m, 2 H, ar-H), 7.80–7.82 (m, 2 H, ar-H) ppm—^13^C-NMR (100 MHz, CDCl_3_): *δ* = 18.11 (Me), 31.91 (2′-CH_2_), 38.07 (1′-CH_2_), 123.37 (ar-CH), 127.08 (ar-CH), 128.34 (ar-CH), 132.28 (ar-Cq), 134.03 (ar-CH), 168.55 (imide CO) ppm—MS (EI, 70 eV): *m*/*z* (%) = 216.1/215.1 [M]^+***·***^ (3/19), 161.1/160.1 [M-C_4_H_7_]^+^ (15/100)—CHN calcd. for C_13_H_13_NO_2_: C: 72.54 H: 6.09 N: 6.51; found C: 72.22 H: 5.99 N: 6.59.

*2-(4*′*-Azidopentyl)isoindoline-1,3-dione (* ***39*** *).* The bromine derivative** 36** (606.3 mg, 2.047 mmol) and NaN_3_ (406.5 mg, 6.253 mmol) were stirred in DMF (dry, 5 mL) at 25°C for 24 hours under nitrogen atmosphere. The solvent was removed with high vacuum and the residue was purified by filtration through a short column of basic alumina (activity level V, DCM 100%) to give** 39** (476.1 mg, 1.843 mmol, and 90%) as a colorless, semisolid compound. Mp 64–66°C (DCM)—IR (ATR-FTIR): $\tilde{\nu }$ = 2933 (w, br), 2097 (m), 1773 (w), 1704 (s), 1614 (w), 1466 (w), 1436 (w), 1395 (m), 1360 (m), 1333 (m), 1267 (w), 1243 (m), 1187 (w), 1171 (w), 1118 (w), 1087 (w), 1048 (m), 1012 (w), 998 (w), 934 (w), 902 (w), 883 (m), 841 (w), 794 (w), 717 (s), 693 (w), 638 (w), 619 (w) cm^−1^—^1^H-NMR (400 MHz, CDCl_3_): *δ* = 1.24 (d, ^3^ *J*_H–H_ = 6.52 Hz, 3 H, Me), 1.46–1.53 (m, 2 H, 3′-CH_2_), 1.66–1.86 (m, 2 H, 2′-CH_2_), 3.43–3.52 (m, 1 H, 4′-H), 3.68 (t, ^3^ *J*_H–H_ = 7.08 Hz, 2 H, 1′-CH_2_), 7.68–7.70 (m, 2 H, ar-H), 7.81–7.83 (m, 2 H, ar-H) ppm—^13^C-NMR (100 MHz, CDCl_3_): *δ* = 19.60 (Me), 25.49 (C-2′), 33.61 (C-3′), 37.73 (C-1′), 57.65 (C-4′), 123.47 (ar-CH), 132.30 (ar-Cq), 134.18 (ar-CH), 168.59 (imide CO) ppm—MS (EI, 70 eV): *m*/*z* (%) = 216.1/215.1 [M-HN_3_]^+***·***^ (4/15), 161.1/160.1 [M-C_4_H_8_N_3_]^+^ (18/100)—CHN calcd. for C_13_H_14_N_4_O_2_: C: 60.45 H: 5.46 N: 21.69; found C: 58.62 H: 4.98 N: 22.28.

## Figures and Tables

**Table 1:** Plain open-chain aliphatic structures with diverse terminal substituents and altered side chain lengths; given bioactivities are against promastigotes of *L*. *major* in comparison to the reference substances miltefosine (**1**) and pentamidine (**2**).

Compound	*n*	R^1^	R^2^	R^3^	R^4^	Bioactivity
**6**	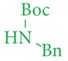					Inactive
**5**	1	Me	Br	Boc	Bn	Active
**7**	1	=CH_2_	H	Boc	Bn	Inactive
**8**	1	Me	N_3_	Boc	Bn	Active
**9**	1	Me	N_3_	H	Bn	Inactive
**10**	1	Me	NH_2_	Boc	Bn	Inactive
**19**	1	H	Br	Boc	Bn	Inactive
**20**	7	H	Br	Boc	Bn	Active
**21**	0	=CH_2_	H	Boc	Bn	Active
**22**	6	=CH_2_	H	Boc	Bn	Inactive
**23**	1	H	N_3_	Boc	Bn	Inactive
**24**	7	H	N_3_	Boc	Bn	Inactive
**25**	1	H	NH_2_	Boc	Bn	Inactive
**26**	7	H	NH_2_	Boc	Bn	Active

**Table 2:** Cyclized substances and heterocyclic structures as terminal substituents with a side chain length of 4 carbon atoms; given bioactivities are against promastigotes of *L*. *major* in comparison to the reference substances miltefosine (**1**) and pentamidine (**2**).

Compound	*n*	R^1^	R^2^	R^3^	R^4^	Bioactivity
**17**		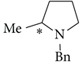				Inactive
**18**		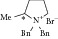				Inactive
**11**	1	Me	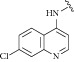	Boc	Bn	Active
**12**	1	Me	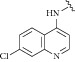	H	Bn	Active
**29**	1	Me	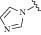	Boc	Bn	Inactive
**30**	1	Me	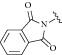	Boc	Bn	Inactive
**14**	1	H	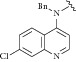	Boc	Bn	Active
**15**	1	H	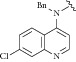	Boc	H	Inactive
**27**	1	Me	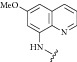	Boc	H	Inactive
**28**	1	Me	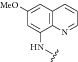	Boc	Bn	Inactive
**40**	1	H	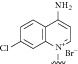	Boc	Bn	Active

**Table 3:** Plain open-chain aliphatic analogs with *N*-benzyl-3,3-dimethylbutanamide functionality; given bioactivities are against promastigotes of *L*. *major* in comparison to the reference substances miltefosine (**1**) and pentamidine (**2**).

Compound	R^1^	R^2^	Bioactivity
**31**	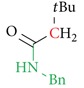		Inactive
**32**	Me	Br	Active
**33**	=CH_2_	H	Not determined
**34**	Me	N_3_	Active
**35**	Me	NH_2_	Inactive

**Table 4:** Pharmacophore analogs with a phthalimide functionality; given bioactivities are against promastigotes of *L*. *major* in comparison to the reference substances miltefosine (**1**) and pentamidine (**2**).

Compound	R^1^	R^2^	Bioactivity
**36**	Me	Br	Inactive
**37**	=CH_2_	H	Inactive
**38**	Me	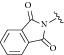	Inactive
**39**	Me	N_3_	Inactive
